# Genome wide association analysis of root hair traits in rice reveals novel genomic regions controlling epidermal cell differentiation

**DOI:** 10.1186/s12870-022-04026-5

**Published:** 2023-01-04

**Authors:** Meredith T. Hanlon, Phanchita Vejchasarn, Jenna E. Fonta, Hannah M. Schneider, Susan R. McCouch, Kathleen M. Brown

**Affiliations:** 1 Department of Plant Science, The Pennsylvania State University, 102 Tyson Building, University Park, PA 16802 USA; 2 Intercollege Graduate Degree Program in Plant Biology, Huck Institutes of the Life Sciences, Penn State University, University Park, PA 16802 USA; 3 ﻿Rice Department, Ministry of Agriculture, Ubon Ratchathani Rice Research Center, Ubon Ratchathani, 34000 Thailand; 4 Centre for Crop Systems Analysis, Wageningen University & Research, Wageningen, the Netherlands; 5 Section of Plant Breeding and Genetics, School of Integrated Plant Sciences, Cornell University, Ithaca, NY 14853-1901 USA; 6 Biological Statistics and Computational Biology, Cornell University, Ithaca, NY 14853-1901 USA

**Keywords:** Root biology, Root hairs, Rice, Subpopulation, Candidate gene, GWAS

## Abstract

**Background:**

Genome wide association (GWA) studies demonstrate linkages between genetic variants and traits of interest. Here, we tested associations between single nucleotide polymorphisms (SNPs) in rice (*Oryza sativa*) and two root hair traits, root hair length (RHL) and root hair density (RHD). Root hairs are outgrowths of single cells on the root epidermis that aid in nutrient and water acquisition and have also served as a model system to study cell differentiation and tip growth. Using lines from the Rice Diversity Panel-1, we explored the diversity of root hair length and density across four subpopulations of rice (*aus*, *indica*, *temperate japonica*, and *tropical japonica*). GWA analysis was completed using the high-density rice array (HDRA) and the rice reference panel (RICE-RP) SNP sets.

**Results:**

We identified 18 genomic regions related to root hair traits, 14 of which related to RHD and four to RHL. No genomic regions were significantly associated with both traits. Two regions overlapped with previously identified quantitative trait loci (QTL) associated with root hair density in rice. We identified candidate genes in these regions and present those with previously published expression data relevant to root hair development. We re-phenotyped a subset of lines with extreme RHD phenotypes and found that the variation in RHD was due to differences in cell differentiation, not cell size, indicating genes in an associated genomic region may influence root hair cell fate. The candidate genes that we identified showed little overlap with previously characterized genes in rice and *Arabidopsis*.

**Conclusions:**

Root hair length and density are quantitative traits with complex and independent genetic control in rice. The genomic regions described here could be used as the basis for QTL development and further analysis of the genetic control of root hair length and density. We present a list of candidate genes involved in root hair formation and growth in rice, many of which have not been previously identified as having a relation to root hair growth. Since little is known about root hair growth in grasses, these provide a guide for further research and crop improvement.

**Supplementary Information:**

The online version contains supplementary material available at 10.1186/s12870-022-04026-5.

## Key message

We present genomic regions associated with root hair length and density in rice. These quantitative traits are potential breeding targets for improved nutrient acquisition.

## Background

Root hairs are small tubular outgrowths of single epidermal cells that contain a number of transporters for nutrient and water acquisition [1]. Early reports indicated that a single rye plant (*Secale cereale* L.) could have up to 14 billion root hairs, providing an additional 400 m^2^ of surface area for soil exploration [2]. This increased surface area is partially due to the small diameter and relatively high surface area to volume ratio of root hairs, especially when compared to larger root structures. The small size of root hairs enables them to penetrate between soil particles to obtain immobile nutrients and improve anchorage and penetration in hard soils and to form a rhizosheath that may improve water acquisition [3–7].

Two separate root hair traits, length and density, increase under low phosphorus (P) conditions in *Arabidopsis* and other species including rice [8–11]. There is substantial genetic variation for these root hair traits in all species studied, and lines with longer and/or denser root hairs have been shown to have greater phosphorus acquisition [12–19]. Under low P conditions, root hairs may contribute up to 90% of total plant P acquisition in *Arabidopsis* [20]. Studies using ^32^P and a root surrounded by mesh that could only be penetrated by root hairs indicated that root hairs contributed 63% of total plant P uptake in barley [14]. Both an increase in the number of root hairs produced closer to the root tip and the increased longevity of an individual hair contribute to increased P acquisition [21, 22]. Since they are considered to be metabolically cheap but highly beneficial [23], root hairs have been identified as a key trait for improving phosphorus acquisition in a number of species [22]. Their utility for water acquisition is less well understood and may be species-specific [24, 25]. Root hairs contribute to rhizosheath formation, which is implicated in drought adaptation [18, 26] and could influence plant–microbe interactions [27, 28]. Root hairs are difficult to phenotype accurately and quickly, but increased root hair length and density traits have been successfully adopted into common bean breeding programs [29]. Recent work has further indicated that root hairs are a promising target for improvement in barley as they improve yield under moisture limiting conditions through improved plant water status and do not confer a yield penalty under optimal conditions [24]. Furthering our understanding of the genetic architecture associated with root hair traits will increase the ability of breeders to incorporate new markers for advantageous root hair growth into selection pipelines.

In addition to their agronomic importance, root hairs have been used as a model system to study differentiation and growth of single cells, since they develop from specific cells of the epidermis [30] and elongate via directed tip growth [31]. The pathways controlling the formation and elongation of root hairs have been studied and reported in *Arabidopsis* [1, 31–45]. The process of epidermal cell patterning and initial hair formation is different in *Arabidopsis* and other Brassicaceae compared to other plant families, including members of the Poaceae [46, 47]. *Arabidopsis* forms longitudinal files of hair and non-hair cells, whereas other plants form hairs over the entire root surface [43, 48–51]. In barley and rice, all epidermal cells have the potential to make a root hair, but cells that eventually form a hair are generally shorter than non-hair forming cells [50, 52]. In other grasses, including *Brachypodium*, the initial division of cells at the root tip is uneven, resulting in a pattern of alternating hair and non-hair cells along the length of the root [50]. Though hundreds of genes have been identified in *Arabidopsis* as players in epidermal cell differentiation [33, 41, 53], little is known about this process in grasses [46, 47].

In rice, several genes have been identified that play a role in root hair elongation, including root hair-specific expansins (*OsEXPB5*, *OsEXPA8,* and *OsEXPA17*) [54, 55], a cellulose synthase gene (*OsCSLD1*) [56], a Sec14-nodulin domain-containing protein (*OsSNDP1*) [57], a formin homology protein (*OsFH1*) [58], three *RBOH* genes [59], a putative cellulase (*RHC*) [60], a *WUSCHEL*-related homeobox gene [61], a NADPH oxidase (*OsNOX3*) [62], and a bHLH transcription factor [63]. The *ROOT HAIR DEFECTIVE-SIX LIKE* (*RSL*) class I genes partially control root hair elongation in *Arabidopsis*, *Brachypodium*, and rice, indicating conservation of some basic mechanisms [64–68]. RSL genes act as master regulators of elongation, though they do not control epidermal cell patterning in grasses [46]. These studies have all leveraged mutants or overexpression lines to identify principal components in the root hair elongation pathway. Mutants that either form ectopic root hairs or have a different mechanism of epidermal cell patterning have not been identified. Transcriptomic profiling of root hairs and root hair forming cells across several dicot and monocot species indicates that the genes controlling root hair patterning in rice and other grasses differ nearly entirely from the well-understood pathways in *Arabidopsis* [69].

To take advantage of the natural diversity in *Oryza sativa*, we undertook a genome wide association (GWA) study to identify genomic regions that control root hair length (RHL) and density (RHD). Leveraging natural variation provides unique insights into the genetics underlying quantitative traits, including RHL and RHD, and can potentially identify genes that were missed or overlooked in mutant screens. We phenotyped a subset (314 lines) of the Rice Diversity Panel-1 (RDP1), a collection of *Oryza sativa* varieties that represent the global diversity of cultivated rice [70, 71]. We then completed GWA analysis using both the High-Density Rice Array (HDRA) [71] and subsets of the rice reference panel (RICE-RP) [72]. The HDRA interrogates 700,000 single nucleotide polymorphisms (SNPs) across all five subpopulations of rice (*indica*, *aus*, *temperate japonica*, *tropical japonica*, and *aromatic*), averaging one SNP every 540 bases. The SNPs are well-distributed across the genome, and the mix of private (subpopulation-specific) and universal SNPs allows for GWA analysis to be performed across and within subpopulations. The imputed SNP set in RICE-RP comprises 4.8 million SNPs, or a SNP every 89 bases, vastly increasing SNP density across the genome for Rice Diversity Panel-1 (RDP1) accessions. We used subsets of the RICE-RP for whole-genome association analysis and the complete set of high-density SNPs for single chromosome analysis. This allows for more detailed examination and interrogation of genomic regions found to be associated with root hair traits in rice while reducing the false-discovery rate.

Using the HDRA in combination with the imputed SNP set, we identified four genomic regions associated with RHL and 14 genomic regions for RHD. The significant SNPs were not linked to genes previously identified to play a role in root hair formation. We re-phenotyped a subset of lines from the initial phenotyping panel based on differing marker profiles within a significant genomic region and discovered that these lines differed in their root epidermal patterning, with more cells elongating into root hairs. This finding indicates that genes within the target genomic region may play a seminal role in rice root epidermal patterning.

## Results

### Association analysis reveals multiple genomic regions related to root hair traits

**Fig. 1:**
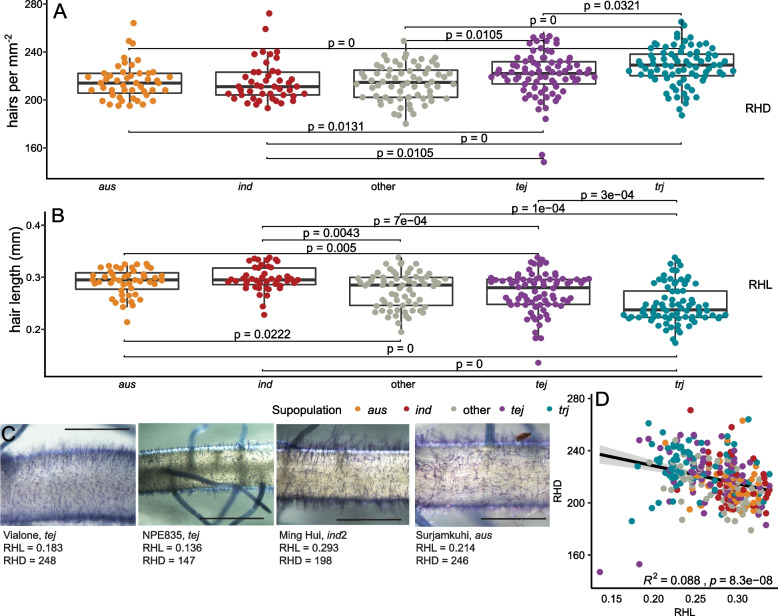
RH traits of all lines. **A** RHD expressed as hairs per mm^2^. The group of lines in “other” includes admixed lines and aromatic lines. Significance is indicated with the displayed *p* value obtained after a Dunn Test. **B** RHL expressed as length in mm. **C** Sample images of four genotypes with their average RHD and RHL across all samples. Scale bar is 1 mm. **D** Correlation between RHD and RHL for all genotypes

Phenotyping of the 314 rice lines for RHL and RHD revealed variation for the traits within and among subpopulations (Fig. 1). *JAPONICA* lines had denser root hairs than *INDICA* lines, with tropical japonica (*trj*) lines having the greatest root hair density (Fig. 1). Length had the opposite pattern, with *trj* lines being the shortest, and *INDICA* lines being longer than *JAPONICA* lines (Fig. 1). Examples of contrasting phenotypes are shown in Fig. 1. RHD and RHL did not show a strong inverse relationship across the whole panel (Fig. 1D). Raw and summarized data are available in Supplemental Tables 1 and 2. Broad-sense heritability ranged from 0.714 to 0.827 (Supplemental Table 2).

**Table 1:** Regions associated with RH traits identified through association analysis

**Region**	**Trait**	**Subpop**	**Chr**	**MS-SNP position**	**MS-SNP** **-log**_**10**_**(*p*)**	**Region size**^a^	**GW assn**^b^	**CW assn.**^c^	**CW gene assn**
D1	RHD	*aus*	1	2,035,050	4.797	158,614	p1, p4, p6	CW	
D2	RHD	*ALL*	1	18,637,547	5.544	219,859	p4, p6, HDRA, GWLD	CW, CWG	LOC_Os01g33869
D3	RHD	*ind*	1	41,918,233	4.819	653,786	p1, p3, p4, p5, p6	CW	
D4	RHD	*ind*	5	1,317,103	5.163	1,783,892	p1, p3, p4, p6	CWG	LOC_Os05g02850
D5	RHD	*ALL*	6	1,510,956	4.781	332,738	p1, p2, p3, p4, p5, p6, HDRA, GWLD	CW, CWG	LOC_Os06g03790
D6	RHD	*ind*	7	21,265,456	4.530	178,997	p3, HDRA, GWLD		
D7	RHD	*ALL*	7	22,510,122	4.903		HDRA, GWLD	CW, CWG	LOC_Os07g37570
D7	RHD	*JAPONICA*	7	23,063,298	4.253	1,713,775	HDRA, GWLD	CW, CWG	LOC_Os07g38410
D7	RHD	*ALL*	7	23,105,291	4.239		HDRA. GWLD	CW, CWG	LOC_Os07g38460
D8	RHD	*ind*	8	19,789,873	6.285	137,470	p1, p3, p4, p6, HDRA, GWLD	CW, CWG	LOC_Os08g32180
D9	RHD	*trj*	8	24,870,099	4.266	639,351	HDRA, GWLD	CW, CWG	LOC_Os08g39350
D10	RHD	*ALL*	9	7,160,663	4.041	33,752	HDRA, GWLD	CW, CWG	LOC_Os09g12500
D11	RHD	*ind*	9	21,413,904	6.039	332,930	p1, p2, p3, p4, p6, GWLD	CW, CWG	LOC_Os09g37500
D12	RHD	*ALL*	10	10,332,255	6.444	1,118,756	p1, p2, p3, p4, p5, p6	CW, CWG	LOC_Os10g20510
D13	RHD	*ind*	12	13,749,121	6.207	352,985	p1, p2, p3, p4, p5, p6, HDRA, GWLD	CW, CWG	LOC_Os12g24140
D14	RHD	*ALL*	12	27,218,429	4.820	97,844	p1, p2, p3, p6	CW, CWG	LOC_Os12g43850
D14	RHD	*JAPONICA*	12	27,256,045	4.826		GWLD	CWG	LOC_Os12g43750
L1	RHL	*ALL*	5	18,292,657	5.506	173,752	p1, p2, p3, p4, p5, p6	CW, CWG	
L2	RHL	*ALL*	6	6,127,858	4.724	188,873	HDRA, GWLD		
L2	RHL	*JAPONICA*	6	6,127,858	4.434		HDRA, GWLD	CW	
L3	RHL	*ALL*	8	2,480,747	5.167	258,148	p1, p2, p3, p4, p5, p6, HDRA, GWLD	CW, CWG	LOC_Os08g04990
L4	RHL	*ALL*	8	7,640,408	4.591	1,001,430	p6, HDRA, GWLD	CW, CWG	LOC_Os08g12790

^a^ Region size was determined by the method in Wang et. al (2018a) [62]

^b^ Genome-wide analyses include the pruned SNP sets from the RICE-RP SNPs (p1-p6), the HDRA SNP set (HDRA), and genome-wide LD blocks from the HDRA set (GWLD)

^c^ Chromosome-wide analysis (CW) includes all RICE-RP SNPs on the respective chromosome. Chromosome-wide gene (CWG) analysis uses the method of Hamazaki & Iwata (2020) [73] to identify associated genes

Using the HDRA and RICE-RP SNP sets, we identified 18 genomic regions related to root hair traits, 14 of which relate to RHD and four to RHL (Table 1, Supplemental Figs. 1-24). No genomic regions were significantly associated with both traits. GWA analysis of individual subpopulations identified genomic regions associated with RHD and RHL that were often distinct from those identified when all lines were used. The “Genome-wide association” and “Chromosome-wide association” columns of Table 1 details which analysis methods identified the association. Regions D7, D9, D10, and L2 were not identified as being significantly associated with any of the pruned SNP sets of the RICE-RP SNPs, but were found using HDRA SNPs, genome-wide LD-block and chromosome-wide gene-based analysis, and chromosome-wide analysis. Regions D7, D14 and L2 were found in both *ALL* and *JAPONICA* analyses. Results from all GWAS runs can be found in the supplemental figures.

Analysis on the chromosome-wide scale using only genic SNPs (with multiple SNPs per gene) resulted in 805 genes being significantly (Bonferroni adjusted *p* < 0.01) associated with RHD (*n* = 514) or RHL (*n* = 277) or both traits (*n* = 14) (Supplemental Table 3). Seventy of the genes were found in previously identified genomic regions, 66 in RHD regions, and four in RHL regions. Two hundred and thirty-one of the genes were previously reported to be expressed in root hairs or root-hair forming cells in rice (Supplemental Table 4).

### Genomic regions controlling root hair length

**Fig. 2:**
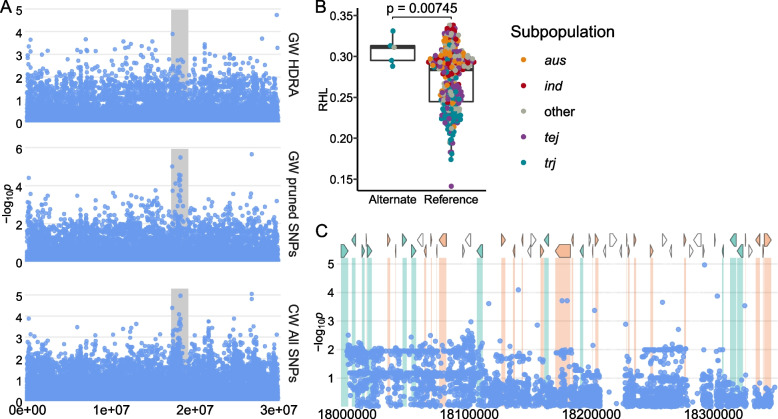
Region L1 on chromosome 5 related to RHL in *ALL*. **A** Chromosome-wide Manhattan plots of genome-wide HDRA (GW HDRA) SNPs (top), a pruned set of genome-wide RICE-RP SNPs (GW pruned SNPs, middle), and all RICE-RP SNPs in a chromosome-wide scan (CW All SNPs, bottom). The grey region indicates the genomic region. **B** Comparison of the RHL phenotype between lines carrying the alternate and reference allele at the MS-SNP. Significance is indicated by a t-test. **C** Magnification of the RICE-RP chromosome-wide Manhattan plot of the genomic region. Genes are overlaid on this plot and colored as follows. Green genes are expressed in root hairs or root-hair forming cells according to Huang et al. [69], orange indicates no previous expression evidence, and white indicates transposable elements

We identified regions associated with RHL on chromosomes 5, 6, and 8 from ALL, and chromosome 6 also from JAPONICA. The region on chromosome 5, L1, was significant using the dense RICE-RP SNP set but not when using the sparser HDRA SNP set. This region is centered around the most significant (MS)-SNP located at 18,292,657 bp (Fig. 2), with a local linkage estimate of 173 kb. The alternate allele confers longer root hairs and is rare; it is found in only four *trj* and one admixed line. We did not find conserved haplotypes in this region (Supplemental Fig. 25). The MS-SNP falls within LOC_Os05g31450, a retrotransposon. The nearest neighboring genes are LOC_Os05g31420, a GRAS family transcription factor, and LOC_Os05g31500, neither of which have previously been identified to expressed in root hairs or root hair forming cells. A full list of genes in this region can be found in Supplemental Table 4.

**Fig. 3:**
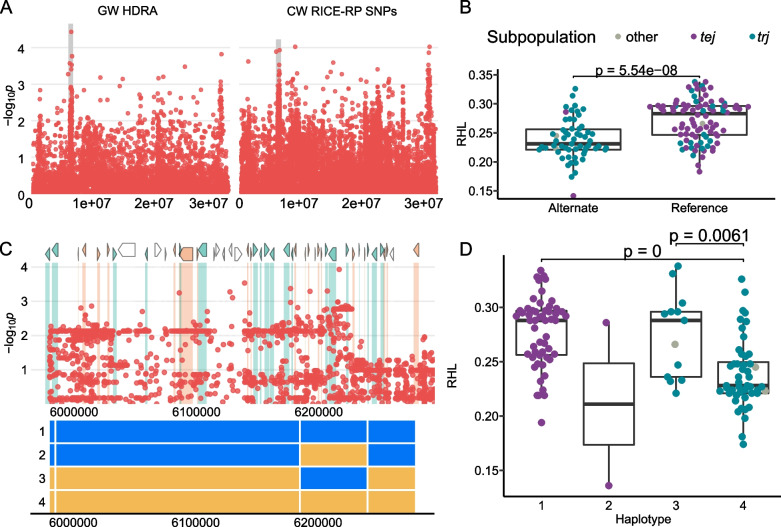
Region L2 on chromosome 6 related to RHL in *ALL* and *JAPONICA*. **A** Chromosome-wide Manhattan plots of HDRA genome-wide analysis (GW HDRA, left) and all RICE-RP SNPs in a chromosome-wide scan (CW RICE-RP SNPs, right). The grey region indicates the genomic region. **B** Comparison of the RHL phenotype between lines carrying the alternate and reference allele at the MS-SNP. Significance is indicated by a t-test. **C** Magnification of the RICE-RP chromosome-wide Manhattan plot of the genomic region. Genes are overlaid on this plot and colored as follows. Green genes are expressed in root hairs or root-hair forming cells according to Huang et al. [69], orange indicates no previous expression evidence, and white indicates transposable elements. Haplotypes are presented below the Manhattan plot, with blue representing reference alleles and yellow, alternate alleles, across these regions. **D** Comparison of the phenotypes of the haplotypes of panel C with significance indicated as a BH-adjusted, Kruskal-Wallace test with a Dunn posthoc test

Region L2 on chromosome 6 was found in both *ALL* and *JAPONICA.* It is approximately 2 Mb in size and is centered around the MS-SNP located at 6,127,858 (Fig. 3) with a local linkage estimate of 189 kb. This region was associated in the HDRA and HDRA-LD block analysis but did not show an association when the imputed SNP set was used (Table 1, Supplemental Figs. 3, 15). Lines carrying the alternate allele had shorter root hairs than those carrying the reference (temperate Japonica (*tej*)) allele, and the majority (*n* = 55) of lines carrying the alternate allele (*n* = 59) were from the *trj* subpopulation (Fig. 3B). Haplotype analysis reveals complete segregation of the two *JAPONICA* subpopulations within this region, with *tej* lines carrying the reference allele as part of Haplotype 1 (H1), and *trj* carrying the alternate allele as part of H2, H3 or H4 (Fig. 3). *Tej* lines carrying H1 have the longest root hairs and *trj* lines carrying H4 have the shortest root hairs (Fig. 3). The MS-SNP is located between two transposons LOC_Os06g11540 and LOC_Os06g11570. A full list of genes in this region can be found in Supplemental Table 4. The gene LOC_Os06g11470, a retrotransposon, had the strongest association in the gene-level analysis.

**Fig. 4:**
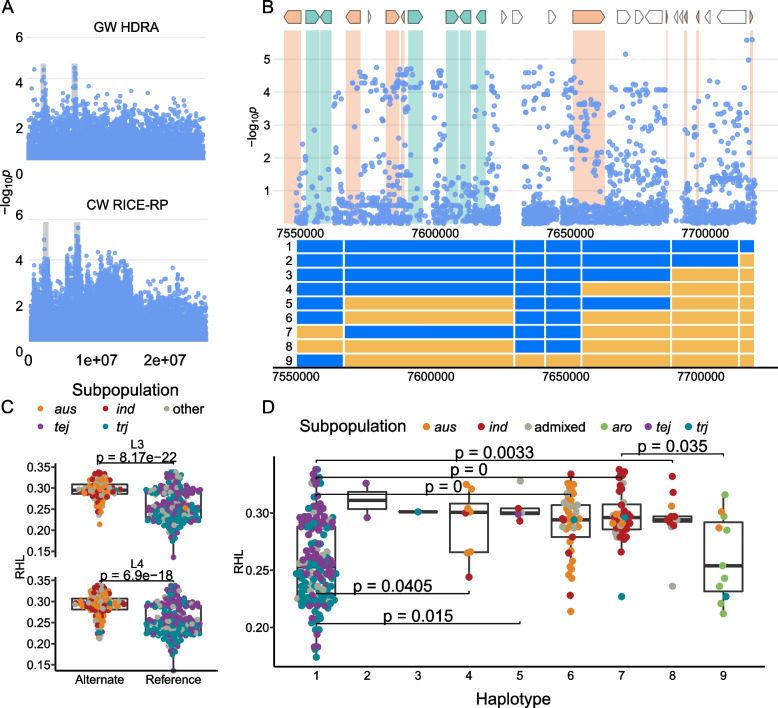
Regions L3 and L4 on chromosome 8 associated with RHL in *ALL*. **A** Chromosome-wide Manhattan plots of HDRA SNPs (top) and all RICE-RP SNPs in a chromosome-wide scan (bottom). The grey region indicates the genomic regions. **B** Magnification of the RICE-RP chromosome-wide Manhattan plot of the genomic region L4. Genes are overlaid on this plot and colored as follows. Green genes are expressed in root hairs or root-hair forming cells according to Huang et al. [69], orange indicates no previous expression evidence, and white indicates transposable elements. Below, haplotypes 1–9 are displayed with blue representing the reference identity and yellow, alternate, across this region. **C** Comparison of the RHL phenotype between ALL lines carrying the alternate and reference allele at the MS-SNP for L3 (top) and L4 (bottom). Significance is indicated by a t-test. **D** Phenotypes of the haplotypes with significance indicated as a BH-adjusted, Kruskal-Wallace test with a Dunn posthoc test

We identified two regions on chromosome 8: L3 is 258 kb in size, centered at 2,598,649 and L4 is 1 Mb, centered at 7,573,725 (Fig. 4). Both regions were significantly associated with RHL in analyses using HDRA SNPs; however, this significance was not clearly maintained throughout and in many of all the runs using the imputed SNP set for region L4 (Table 1, Supplemental Fig. 3). Lines carrying the alternate allele at both the L3 and L4 MS-SNPs had longer root hairs than lines carrying the reference SNP (Fig. 4). The alternate allele is carried by a majority of *aus, ind,* and admixed lines at both loci. The presence of the alleles in a majority of *INDICA* lines is consistent with the fact that *INDICA* varieties have longer root hairs on a population scale (Fig. 1) as compared to *JAPONICA* varieties. Genes in both of these regions are presented in Supplemental Table 4. No *JAPONICA* lines carry the alternate allele at the MS-SNP in L3. Lines carrying the alternate allele at the L4 MS-SNP centered at 7,573,725 (*n* = 141) include all *ind* (*n* = 72) and *aus* (*n* = 47) lines and a minority of *tej* (*n* = 4) and *trj* (*n* = 5) lines. Lines carrying the alternate allele at this SNP have longer root hairs than those carrying the reference allele (Fig. 4). We identified nine haplotypes across this region that segregate by subpopulation (Fig. 4). Haplotypes 1–3 are found predominantly in *JAPONICA* varieties and are associated with shorter root hairs while Haplotypes 4–8 are found predominantly in *INDICA* varieties associated with longer root hairs. Haplotype H9 is found predominantly in *aro* varieties and is associated with shorter root hairs than the other lines carrying alternate alleles at the L4 MS-SNPs. Candidate genes in this region include four genes previously linked to root hair formation—LOC_Os08g12680, a zinc finger domain, LSD subclass family protein, LOC_Os08g12750, a serine-threonine protein kinase, LOC_Os08g12820, a proteasome/cyclosome repeat containing protein, and LOC_Os08g12830, a cytidylyltransferase domain containing protein. A full list of genes can be found in Supplemental Table 4.

### Genomic regions controlling root hair density

We identified 14 regions associated with RHD on chromosomes 1, 5, 6, 7, 8, 9, 10, and 12 (Table 1). Four of these regions were identified in *ALL* lines (chromosomes 1, 6, 9, and 10), two were identified in *ALL* and *JAPONICA* (chromosomes 7 and 12)*,* one was uniquely identified in *aus* (chr 1), one was uniquely identified in *trj* (chr 8) and regions on six chromosomes were uniquely identified in *ind* (Table 1). Regions D4 (*ind* chromosome 5, Supplemental Fig. 26), D7 (*ALL* and *JAPONICA* chromosome 7, Supplemental Fig. 27), D9 (*trj* chromosome 8, Supplemental Fig. 28), and D10 (*ALL* chromosome 9, Supplemental Fig. 29) lacked support using the RICE-RP SNPs in either the pruned GWA or chromosome-wide analysis and were not examined further. Genes located in these regions are listed in Supplemental Table 4.

**Fig. 5:**
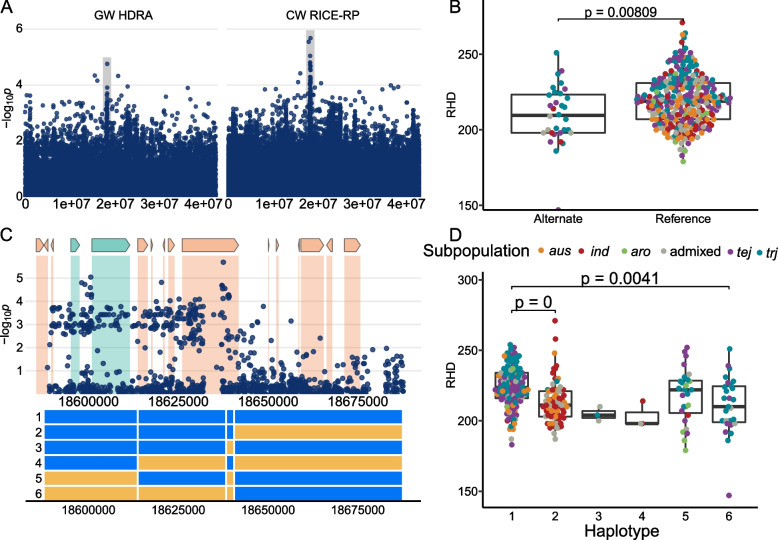
Region D2 on chromosome 1 related to RHD in *ALL* **A** Chromosome-wide Manhattan plots of HDRA SNPs (left) and all RICE-RP SNPs in a chromosome-wide scan (right). The grey region indicates the genomic region. **B** Comparison of the RHD phenotype between ALL lines carrying the alternate and reference allele at the MS-SNP. Significance is indicated by a t-test. **C** Magnification of the RICE-RP chromosome-wide Manhattan plot of the genomic region. Genes are overlaid on this plot and colored as follows. Green genes are expressed in root hairs or root-hair forming cells according to Huang et al. [69], orange indicates no previous expression evidence, and white indicates transposable elements. Below, haplotypes 1–6 are displayed with blue representing the reference identity and yellow, alternate, across this region. **D** Phenotypes of the haplotypes with significance indicated as a BH-adjusted, Kruskal-Wallace test with a Dunn-posthoc test

Three distinct regions on chromosome 1 were identified in *ALL, ind*, and *aus*. Region D1 (*aus*) is 158 kb in size and centers around the MS-SNP located at 2,035,050 bp (Supplemental Fig. 30). This region was not identified using the HDRA SNPs but was detected in multiple genome-wide scans using the RICE-RP SNP set (Table 1). This is a rare allele (*n* = 3) found only in the *aus* subpopulation that results in denser root hairs. A list of the genes in this region can be found in Supplemental Table 4; no gene-level association was identified in our analysis (Supplemental Fig. 30). Region D2 detected in *ALL* is 219 kb in size and centers around the MS-SNP located at 18,637,547 bp (Fig. 5). The alternate allele is found in a total of 32 lines representing the *tej* (8), *trj* (17), and *ind* (3) subpopulations (Fig. 5) and is associated with less dense root hairs. The three *ind* lines carrying the alternate allele (China 1039, Yodanya, and JCC117) all belong to the *ind1* subgroup. We identified six major haplotypes across the D2 region (Fig. 5). Haplotype 1 is found in most *JAPONICA* lines and is associated with high root hair density, a trait considered favorable for nutrient acquisition. H2 is found in a majority of *INDICA* lines, and along with H3 and H4, which are rare haplotypes, is associated with lower RHD. H5, which is carried by *JAPONICA* and a few *aromatic* lines has relatively high RHD, while H6 is a *JAPONICA-*specific haplotype that results in low RHD (Fig. 5). Two nearby genes in this region, LOC_Os01g33784 and LOC_Os01g33800, are expressed in root hairs. The MS-SNP falls within LOC_Os01g33869, a gene not expressed in root hairs.

**Fig. 6:**
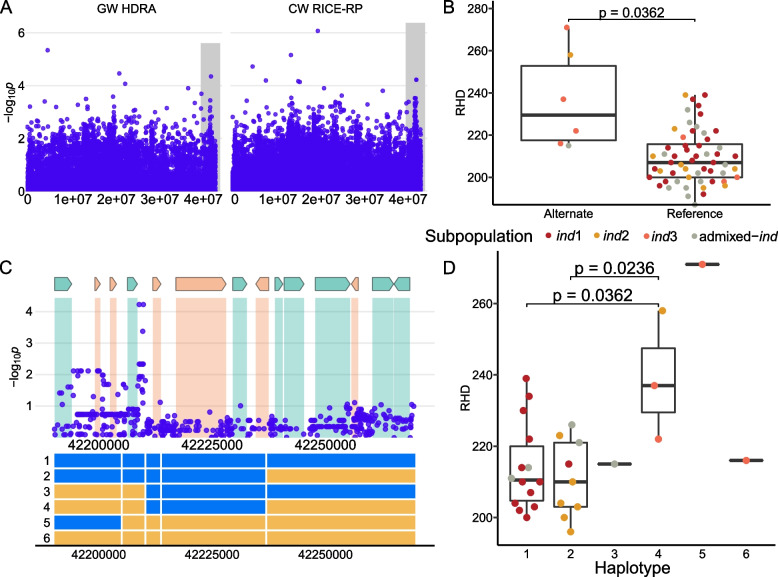
Region D3 on chromosome 1 related to RHD in *ind*. **A** Chromosome-wide Manhattan plots of HDRA SNPs (left) and all RICE-RP SNPs in a chromosome-wide scan (right). The grey region indicates the genomic region. **B** Comparison of the RHD phenotype between *ind* lines carrying the alternate and reference allele at the MS-SNP. Significance is indicated by a t-test. **C** Magnification of the RICE-RP chromosome-wide Manhattan plot of the genomic region. Genes are overlaid on this plot and colored as follows. Green genes are expressed in root hairs or root-hair forming cells according to Huang et al. [69], orange indicates no previous expression evidence, and white indicates transposable elements. Below, haplotypes 1–6 are displayed with blue representing the reference identity and yellow, alternate, across this region. **D** Phenotypes of the haplotype with *p* values from a Dunn posthoc test

The D3 *ind* region is 654 kb in size and centers around the MS-SNP located at 41,918,233 bp and was not identified using the HDRA SNPs (Fig. 6). Five lines (RTS4 (Vietnam), Ming Hui (China), SL 22–613 (Sierra Leone), Djimoron (Guinea), Co18 (India), and SLO17 (India)) carry the alternate allele at the MS-SNP and have significantly denser root hairs (Fig. 6B). These lines come from both the *ind2* and *ind3* subgroups. Haplotype analysis of this region reveals six haplotypes, one of which is rare and results in denser root hairs (H4). H4 is found in only three varieties, Ming Hui, Djimoron, and SL 22–613. Of the 93 genes in this region, 44 have previously been found to be expressed in root hairs or root hair forming cells. The MS-SNP is nearest to LOC_Os01g72740, a cytochrome p450 expressed in root hairs. All genes in this region can be found in Supplemental Table 4.

**Fig. 7:**
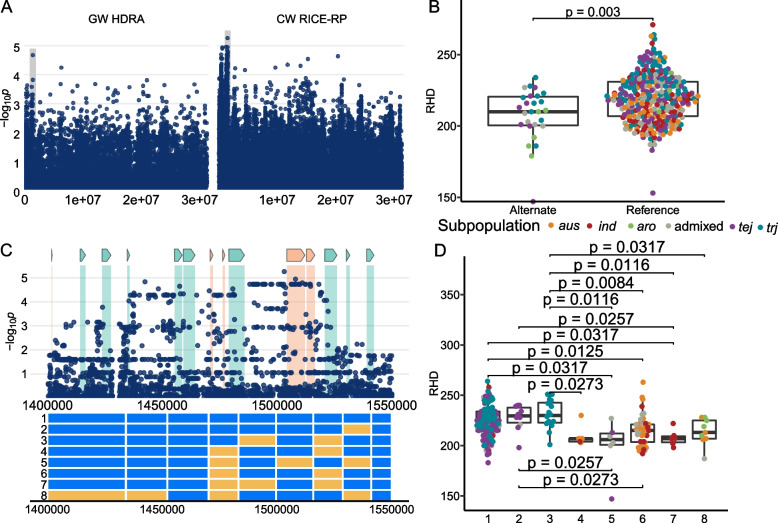
Region D5 on chromosome 6 related to RHD in *ALL* **A** Chromosome-wide Manhattan plots of HDRA SNPs (left) and all RICE-RP SNPs in a chromosome-wide scan (right). The grey region indicates the genomic region. **B** Comparison of the RHD phenotype between *ALL* lines carrying the alternate and reference allele at the MS-SNP. Significance is indicated by a t-test. **C** Magnification of the RICE-RP chromosome-wide Manhattan plot of the genomic region. Genes are overlaid on this plot and colored as follows. Green genes are expressed in root hairs or root-hair forming cells according to Huang et al. [69], orange indicates no previous expression evidence, and white indicates transposable elements. Below, haplotypes 1–8 are displayed with blue representing the reference identity and yellow, alternate, across this region. **D** Phenotypes of the haplotypes with significance indicated as a BH-adjusted, Kruskal-Wallace test with a Dunn posthoc test

Region D5 is 332 kb in size and was identified in *ALL* lines. It lies on chromosome 6 and lines carrying the alternate allele at the MS-SNP located at 1,510,956 bp have less dense root hairs (Fig. 7). The alternate allele is found only in lines from the *JAPONICA* and *aro* subpopulations, though this region was not significantly associated with RHD in the *JAPONICA*-specific clade analysis. We identified eight haplotypes across the D5 region with four or more lines in each (Fig. 7). H1, H2 and H3 were carried by *JAPONICA* varieties and had higher RHD that H4-H8, which were carried predominantly by *INDICA* and *aromatic* varieties. There are 59 genes in this region, 20 of which have root-hair related expression. The gene closest to the MS-SNP, LOC_Os06g03780, a NUC153 domain containing protein, has no root-hair elements in its promoter, nor is it expressed in root hairs or hair-forming cells.

**Fig. 8:**
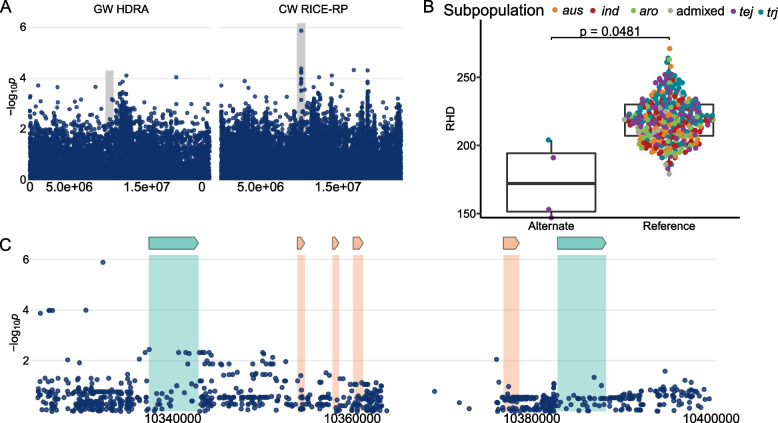
Region D12 on chromosome 10 related to RHD in *ALL* **A** Chromosome-wide Manhattan plots of HDRA SNPs (left) and all RICE-RP SNPs in a chromosome-wide scan (right). The grey region indicates the genomic region. **B** Comparison of the RHD phenotype between *ALL* lines carrying the alternate and reference allele at the MS-SNP. Significance is indicated by a t-test. **C** Magnification of the RICE-RP chromosome-wide Manhattan plot of the genomic region. Genes are overlaid on this plot and colored as follows. Green genes are expressed in root hairs or root-hair forming cells according to Huang et al. [69] and orange indicates no previous expression evidence

Two additional regions were identified in *ALL* on chromosomes 10 and 12 (D12 and D14, respectively), with the region on chromosome 12 also appearing in the *JAPONICA*-specific analysis. Region D12 is 1.1 Mb in size and is centered around the MS-SNP at 10,332,255 bp. The alternate allele is a rare, *JAPONICA* specific variant that results in less dense root hairs (Fig. 8). Three of the four lines carrying the minor allele are from *tej* (NPE 835, Hatsunishiki, and Kamenoo) and one from *trj* (KU 115). The MS-SNP is upstream of LOC_Os10g20510, a gene expressed in both root hairs and root-hair forming cells. Other genes in this region can be found in Supplemental Table 4. There were no conserved haplotypes or evidence of introgression in this region (Supplemental Fig. 31). Region D14 is 98 kb in size and was found on chromosome 12 in *ALL* and *JAPONICA* with the MS-SNP located at 27,218,429 bp (Supplemental Fig. 32). The alternate allele is specific to the *trj* subpopulation and is associated with less dense root hairs. Two lines with extremely sparse root hairs are driving the association, as the mean values of the two genotypic groups do not differ when these are removed. We did not explore this region further, but information can be found in Supplemental Table 4 and Supplemental Fig. 32.

**Fig. 9:**
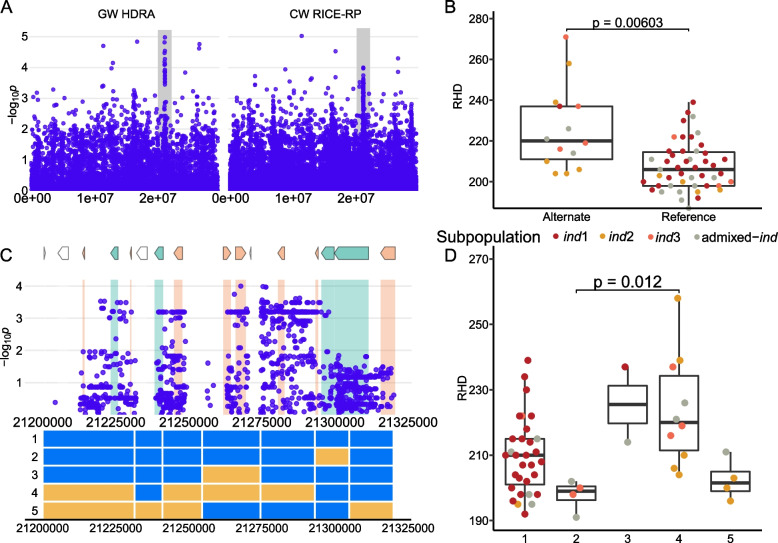
Region D6 on chromosome 7 related to RHD in *ind* **A** Chromosome-wide Manhattan plots of HDRA SNPs (left) and all RICE-RP SNPs in a chromosome-wide scan (right). The grey region indicates the genomic region. **B** Comparison of the RHD phenotype between *ind* lines carrying the alternate and reference allele at the MS-SNP. Significance is indicated by a t-test. **C** Magnification of the RICE-RP chromosome-wide Manhattan plot of the genomic region. Genes are overlaid on this plot and colored as follows. Orange genes are not expressed in root hairs or root-hair forming cells according to Huang et al. [69]. Below, haplotypes 1–5 are displayed with blue representing the reference identity and yellow, alternate, across this region. **D** Phenotypes of the haplotypes with significance indicated as a BH-adjusted, Kruskal-Wallace test with a Dunn posthoc test

**Fig. 10:**
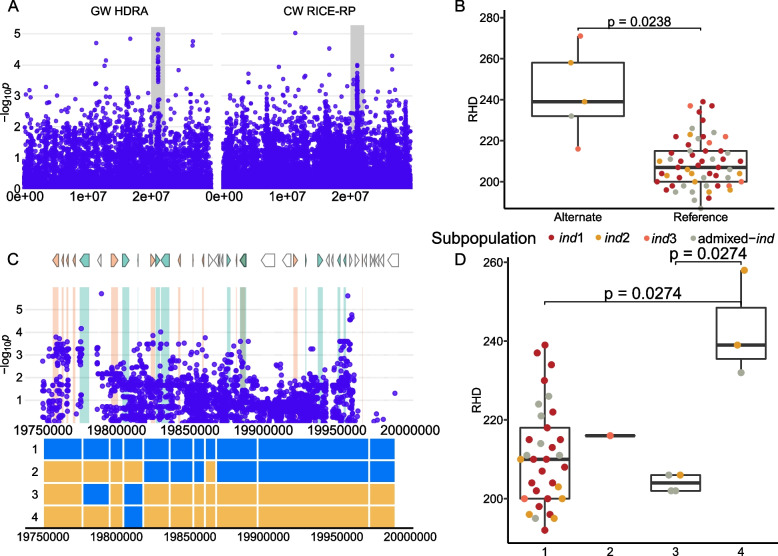
Region D8 on chromosome 8 related to RHD in *ind* **A** Chromosome-wide Manhattan plots of HDRA SNPs (left) and all RICE-RP SNPs in a chromosome-wide scan (right). The grey region indicates the genomic region. **B** Comparison of the RHD phenotype between *ind* lines carrying the alternate and reference allele at the MS-SNP. Significance is indicated by a t-test. **C** Magnification of the RICE-RP chromosome-wide Manhattan plot of the genomic region. Genes are overlaid on this plot and colored as follows. Green genes are expressed in root hairs or root-hair forming cells according to Huang et al. [69], orange indicates no previous expression evidence, and white indicates transposable elements. Below, haplotypes 1–4 are displayed with blue representing the reference identity and yellow, alternate, across this region. **D** Phenotypes of the haplotypes with significance indicated as a BH-adjusted, Kruskal-Wallace test with a Dunn posthoc test

Four regions associated with root hair density were found on chromosomes 7, 8, 9, and 12 (D6, D8, D11, and D13) in *ind*. Alternate alleles at the MS-SNPs in each region were associated with denser root hairs. The MS-SNP in region D6 is centered at 21,265,456 bp, and 14 lines carry the alternate allele (Fig. 9). The region is 178 kb in size and there are only two genes in the immediate vicinity of the MS-SNP—LOC_Os07g35530 and LOC_Os07g35540, neither of which are expressed in root hairs or root-hair forming cells. Haplotype 1, the predominant haplotype in the D6 region, is found predominantly in *ind1* lines, H2 is carried by 4 lines and is associated with lower RHD, H3 and H4 are associated with higher RHD and are found across all subsets of *ind* lines, including admixed lines, and H5, a rare haplotype carried mostly by *ind2* lines is associated with lower RHD, similar to H2 (Fig. 9). Region D8 is 137 kb in size and is centered at the MS-SNP located at 19,789,873 bp on chromosome 8 (Fig. 10). Five lines (RTS4, Ming Hui, Tadukan, Co18, and Sigadis) carry the minor allele at the D8 MS-SNP (Fig. 10). Haplotype analysis reveals four haplotypes, with H1 being the predominant haplotype at this locus and associated with low RHD, and H4, a rare but conserved haplotype carried by Ming Hui, Co18, and Sigadis, is associated with denser root hairs. The genomic variation that distinguishes H4 (high RHD) from H3 (low RHD) in our analysis occurs in a region that contains no genes. Thus, we cannot draw any conclusions about potential causal genes in this region. A full list of genes across the D8 region can be found in Supplemental Table 4.

**Fig. 11:**
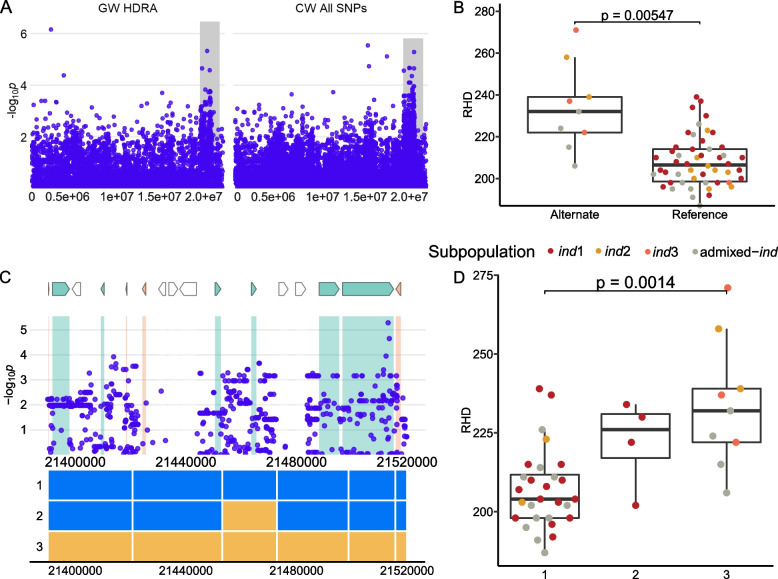
Region D11 on chromosome 9 related to RHD in *ind* **A** Chromosome-wide Manhattan plots of HDRA SNPs (left) and all RICE-RP SNPs in a chromosome-wide scan (right). The grey region indicates the genomic region. **B** Comparison of the RHD phenotype between *ind* lines carrying the alternate and reference allele at the MS-SNP. Significance is indicated by a t-test. **C** Magnification of the RICE-RP chromosome-wide Manhattan plot of the genomic region. Genes are overlaid on this plot and colored as follows. Green genes are expressed in root hairs or root-hair forming cells according to Huang et al. [69], orange indicates no previous expression evidence, and white indicates transposable elements. Below, haplotypes are displayed with blue representing the reference identity and yellow, alternate, across this region. **D** Phenotypes of the haplotypes with significance indicated as a BH-adjusted, Kruskal-Wallace test with a Dunn posthoc test

Region D11 is centered around the MS-SNP located at 21,513,108 bp on chromosome 9 (Fig. 11). Lines carrying alternate alleles across this 130 kb region containing the MS-SNP had denser root hairs. No *ind1* subgroup lines carry the alternate allele at the MS-SNP. The MS-SNP is located within LOC_Os09g37240, a glutathione S-transferase expressed in root-hair forming cells. The change in the MS-SNP from its reference identity, C, to its alternate identity, G, results in a predicted nonsynonymous mutation within exon 15 of 19, resulting in a shift from arginine to serine, and the gene contains a root-hair element cis-factor in the promoter. In the analysis using genic SNPs, this gene was not significantly associated with RHD. Other genes in this region include LOC_Os09g37230, a kinase expressed in hairs and cells, LOC_Os09g37200, a transferase expressed in hairs and cells, LOC_Os09g37100, a phospholipase expressed in hairs and cells, and LOC_Os09g37080, also expressed in hairs and cells. Lines carrying the alternate haplotype were Ming Hui, SL 22–613, Djimoron, Tadukan, Seratoes Hari, Co 18, Sigadis, Chiem Chanh, and Slo 17.

**Fig. 12:**
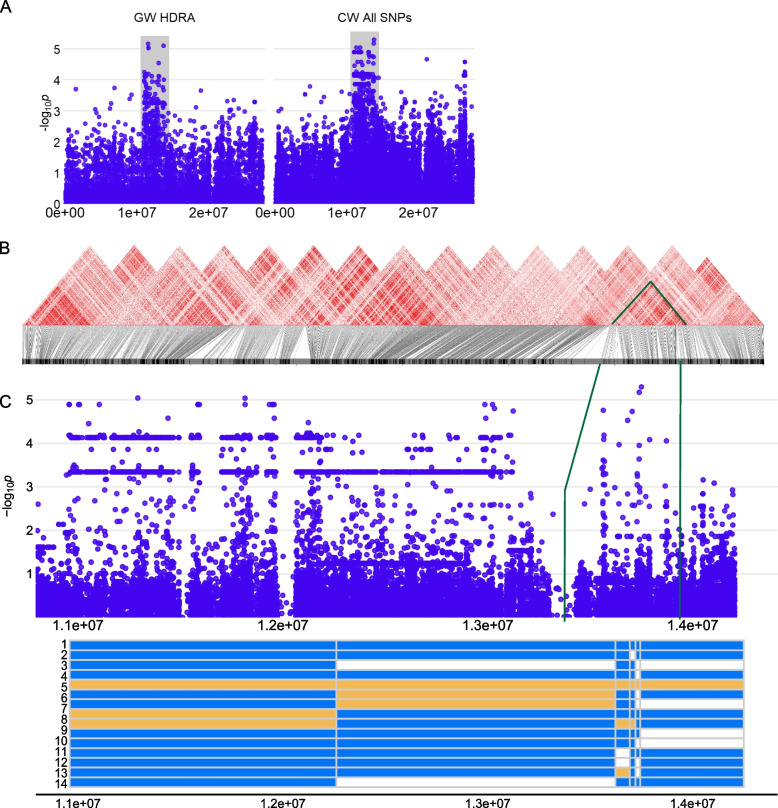
Region D13 on chromosome 12 related to RHD in *ind* **A** Chromosome-wide Manhattan plots of HDRA SNPs (left) and all RICE-RP SNPs in a chromosome-wide scan (right). The grey region indicates the genomic region. **B** r2 based linkage analysis of the SNPs in this region. SNPs were randomly thinned and included in linkage analysis. Stronger linkage is represented by red coloring. A linkage block around the MS-SNP is outlined in green. **C** Magnification of the RICE-RP chromosome-wide Manhattan plot of the genomic region. Below, haplotypes are displayed with blue representing the reference alleles and yellow, alternate, across this region. Heterozygotes or missing reads are displayed as white

We identified a large 3 Mb region on chromosome 12 (D13) in *ind* lines. The MS-SNP is located at 13,749,121 bp, close to the centromeric region on chromosome 12 (11,873,007 to 12,010,077 bp) (Fig. 12), which may, in part, explain the large linkage block observed in the SNPs. We also observed many heterozygous or missing reads in this region, particularly around the MS-SNP, suggesting potential structural variation and giving rise to many haplotypes. Examination of the *r*^*2*^ linkage plots reveals smaller regions of linkage around the MS-SNP and in upstream regions (Fig. 12). Two lines, Ming Hui and Co 18 (H5), carry the alternate allele at the MS-SNP and four additional lines, RTS4, SL 22–613, Bala, and Sigadis, carry alternate alleles other SNPs in the MS-SNP region (H6-9). Due to the extensive linkage disequilibrium and the large number of significant SNPs in the region, we cannot confidently identify specific causal genes for D14. All genes, however, are presented in Supplemental Table 4.

### Candidate gene comparison with known root hair genes

Relatively few genes with known root hair-related functions have been identified in grasses, with more genes identified in rice than in other members of the family. Most genes related to root hair formation have been first identified in *Arabidopsis*, which has a different cell differentiation pattern than the grasses, making the conservation of mechanisms unlikely. To investigate the relationship between genes identified in this study and those identified previously, we first compared our list of candidate genes within significant GWA regions with a list of genes previously identified as having a role in root hair formation in rice (Supplemental Table 5). None of the genes in our associated regions have previously been identified to be related to root hair growth or formation in rice. None of the genes in our study belonged to orthologous groups of core *Arabidopsis* root hair genes [33]. Of the 700 genes we identified in all of the root-hair associated regions (Supplemental Table 4), 140 were found to be expressed in both root hairs and root-hair forming cells, 57 in cells only, and 28 in hairs only [69]. Ten of these expressed genes were also found to be expressed in a different study [74]. Regions L3 and L4 both overlap with a previously identified QTL related to RHD that spans from 249,551 to 8,750,000 [75].

### Candidate genes with *cis*-acting root hair elements (RHEs)

Using the MEME suite tool FIMO, we identified 217 genes with root hair elements (RHEs) in their putative promoter regions (Supplemental Table 6). The RHE sequence is a 16 or 17 bp long sequence found in many genes related to root hair formation in *Arabidopsis* [76]. Of these, 56 are expressed in both root hairs and root-hair forming cells, 16 in hairs, and 22 in root-hair forming cells only [69], indicating an imperfect relationship between RHE presence and measured expression.

### Regions from *ind* control root hair density through cell differentiation

**Fig. 13:**
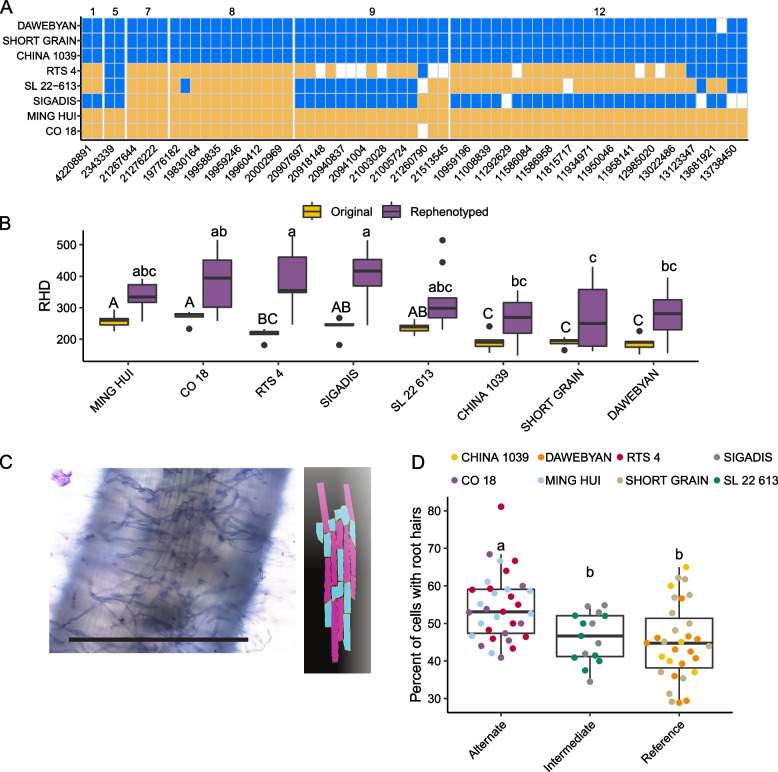
Phenotyping of a select set of lines to investigate controls of RHD. **A** Significant SNPs from our analyses if *ind* lines across chromosomes 1, 5, 7, 8, 9, and 12. The lines we selected to phenotype further carried either the allele identity of reference (blue), alternate (yellow), or indeterminate (white). **B** Lines differ in density between the original (yellow, nodal roots, 8-week-old plants) and rephenotyped data sets (purple, seminal roots, 3-week-old plants). **C** Micrograph of the surface of a rice root with processed image highlighting hair (blue) and non-hair cells (pink). Scale bar represents 1 mm. **D** Lines do differ in the percent of cells that differentiate into hair forming cells. Significance was determined by a Tukey HSD test *p* < 0.05

To further investigate the mechanism by which genomic regions identified in this study modulate RHD, we re-phenotyped a subset of *ind* lines carrying favorable alleles across multiple sites described above associated with denser root hairs, a favorable phenotype for nutrient acquisition. In the initial experiments, phenotyping was done on 8-week-old plants while in this experiment 3-week-old plants were used, and results were much more variable. Two of the lines used for re-phenotyping, Ming Hui and CO 18, carry alternate alleles at all significantly associated SNPs on chromosomes 1, 5, 7, 8, 9, and 12 (Fig. 13). Three other lines, RTS 4, SL 22–613, and Sigadis, carry alternate alleles at SNPs on at least three of these chromosomes. We compared the detailed phenotypes of these five varieties (Fig. 13) to three lines that carry reference alleles at all associated SNPs – China 1039 (China), Short Grain (Thailand), and Dawebyan (Myanmar). Two genotypes, Sigadis and RTS4, had significantly denser root hairs (Tukey HSD, *p* < 0.05), than the sparse lines (China 1039, Short Grain, and Dawebyan) (Fig. 13). We also measured individual cell dimensions (Fig. 13) and observed no significant differences in the total number of cells per mm^2^ (data not shown). Using the SNP identities in Fig. 13A, we grouped the lines into three groups: those carrying a majority of alternate alleles (Ming Hui, CO 18, and RTS4), those with an intermediate genotype (Sigadis and SL 22–613), and those with reference alleles (China 1039, Short Grain, and Dawebyan). We compared the percent of cells along the root epidermis that differentiated into root hairs and found the lines with a majority of alternate alleles differentiated more cells into root hairs compared to those with intermediate or reference genotypes (Fig. 13). The lines in the alternate group differentiated an average of 54.5% of their cells into root hairs, Ming Hui (54.7%), (CO 18 (51.2%), and RTS 4 (56.1%), whereas lines carrying all reference alleles differentiated an average of 45.1% (China 1039 (46.9%), Short Grain (47.0%), and Dawebyan (41.6%)) (Fig. 13). The intermediate genotypes differentiated 46.3% of their cells into root hairs (Sigadis (47.6%) and SL 22 613 (45.1%)). This suggests that genes or genomic regions identified in this study may control the process of cell differentiation and not merely cell density or root growth.

## Discussion

Root hairs have been studied as both a target trait for crop improvement and as a system to understand cellular patterning, differentiation, and growth. Our understanding of root hair development is largely limited to *Arabidopsis*, a Brassicaceae species, which has a root hair patterning mechanism that differs greatly from other plant families, especially grasses. Here, we used natural variation and linkage analysis to identify genomic regions and candidate genes potentially related to root hair length and density in rice. Many of the candidate genes that we discovered have not been previously identified as playing a role in root hair formation or growth. We present a condensed list of 43 candidate genes in Supplemental Table 7. These genes that are significantly associated with RHL or RHD when GWA analysis is performed using both dense sets of anonymous SNPs and when using genic SNPs alone. They are expressed in root hairs or root-hair forming cells or have a *cis*-acting RHE in their putative promoter sequence. We include LOC_Os09g37240 in this list due to the SNP-level evidence described above.

We integrated previous studies [69, 74] profiling gene expression in root hairs and root hair forming cells into our analysis. Lack of expression in root hair cells, however, does not necessarily preclude a gene from involvement in root hair formation. Root epidermal patterning in *Arabidopsis* is largely controlled through a series of transcriptional feedback loops, resulting in the phenotype of hair and non-hair cells [34, 77–81]. Proper expression, regulation, and movement of transcription factors and transcription factor complexes is necessary for normal epidermal cell differentiation of both hair and non-hair cells in *Arabidopsis*. It is unknown whether these same negative feedback loops control epidermal cell patterning in rice, and whether genes expressed in root-hair forming cells result in root hair formation or if genes expressed in non-hair cells repress the formation of root hairs. Recent work has shown that less severe mutations of a key player in cell differentiation, *TTG1*, results in significantly altered root hair patterning of the *Arabidopsis* epidermis [82], suggesting that knockout and other severe mutants may not provide meaningful insight into the roles that these genes play in the pathway. Though tissue-specific expression profiles have been generated for genes transcribed in rice root hairs, our lack of knowledge about the specific genes controlling epidermal cell differentiation makes it impossible to determine if specific genes could potentially be involved in regulatory mechanisms controlling root hair differentiation. GWA allows us to use natural variation to narrow down the list of genes that may be involved in a process like root hair formation without altering the genome or introducing epistatic effects. Tools to combine expression network data with GWA results have been developed to prioritize candidate genes obtained from GWA studies [83, 84]; however, we did not find a relationship between previously published root-hair data [69] and our GWA results. Here, we present both candidate genes and genomic regions that merit further investigation for their involvement in root hair formation and growth.

Previous research based on biparental mapping (Aus276 x IR64) identified a region on chromosome 8 that was significantly associated with root hair length, measured via a rank-scoring method in field-grown plants [75]. Plants carrying the favorable (Aus276) QTL alleles had root hairs that were 20–40% longer than those carrying the alternate QTL. This QTL coincides with genomic regions L3 and L4 identified in this study. There are twenty-six genes in the L3 and L4 regions that are expressed in root hairs or root-hair forming cells. None of these genes have previously been identified as playing a role in root hair formation. A single gene, LOC_Os08g12820, a proteasome/cyclosome repeat containing protein, was significant in the gene-level analysis, is expressed in both cells and hairs, and contains a cis-acting RHE in its putative promoter region. The *Arabidopsis* ortholog of this gene, AT2G32730, RPN2a, is differentially phosphorylated under auxin application, indicating a possible role in root hair growth [85]. This suggests that LOC_Os08g12820 warrants further investigation into its role in root hair growth.

Of the 14 genomic regions associated with RHD in this study, six were identified based on variation in the *indica* subpopulation and six from the diversity panel as a whole (*ALL*). Two of the regions identified in *ALL* were also significant in the clade-level *JAPONICA* analysis. The *ind* lines carrying the largest number of favorable alleles were from diverse geographic regions. Ming Hui (China) and Sigadis (Indonesia) are classified as belonging to the *ind2* subgroup and Co18 (India), RTS4 (Vietnam), and SL 22–613 (Sierra Leone) are *ind3* lines [72].

We identified LOC_Os09g37240, a glutathione S-transferase expressed in root-hair forming cells in region D11 with a non-synonymous mutation that may alter protein function that is expressed in root-hair forming cells and contains a RHE in its putative promoter region. This gene, also known as Os09g0544400, has been previously shown to be associated with rice grain weight [86], though there is no evidence of its important in root hair formation or development. *Arabidopsis* glutathione S-transferases have been shown to be up-regulated by ethylene and play a role in early epidermal patterning [87], lending evidence that this gene may play a role in rice root epidermal patterning. The large, 3 Mb region that we identified on chromosome 12 contains 11 genes that may warrant further investigation. Of these, two genes, LOC_Os12g18880 and LOC_Os12g24080, are both expressed in root hairs and root-hair forming cells and contain a RHE *cis* element in their promoter region. LOC_Os12g18880 is *OsMAGO2*, which is involved in multiple developmental pathways [88]. *Arabidopsis* plants with a mutation in a Mago ortholog have altered root hair differentiation patterns [89]. LOC_Os12g24080, also known as LARGE2 or *OsUPL2*, encodes a HECT-domain E3 ubiquitin ligase and has been shown to be related to panicle size and grain number [90]. Though not shown to be related to root-hair formation previously, HECT-domain E3 ligases are related to trichome differentiation, a process that shares many regulatory similarities with root hair formation [91].

## Conclusions

In this study, we present a list of genomic regions and candidate genes for two quantitative traits, root hair length and root hair density. These traits have complex and independent genetic control in rice. Many of the genes we present as candidates have not been previously identified as having a relation to root hair growth. Exploiting natural variation, complemented by other studies using induced mutations, provides us with new insight into mechanisms that may control root hair formation. Root hairs are both critical to plant performance and serve as a basic model for understanding cellular patterning and tip growth. The genes and genomic regions presented here can be productively investigated as targets for selection, to further our knowledge about root hair growth and grasses, and to deepen our understanding of basic plant biology.

## Methods

### Phenotyping

A selection of 314 lines of the Rice Diversity Panel-1 (RDP-1, available from the USDA-ARS, Dale Bumpers National Rice Research Center, Stuttgart, Arkansas, USA, Supplemental Table 1 [70, 71]) were phenotyped for RHL and RHD after being grown in the greenhouse at Pennsylvania State University, University Park, PA (40º49’ N, 77º52’ W). Plants were grown in a volume-based mixture of medium-sized, commercial-grade sand (40%, Quickrete Companies, Inc., Harrisburg, PA), horticultural vermiculite (59%, Whittemore Companies, Inc., Lawrence, MA, USA), and solid-phased buffered phosphorus (1%) prepared according to [92] to provide sufficient, constant phosphate (100 μM) to the growing plants. Plants were irrigated daily with Yoshida nutrient solution [93] at a pH of 5.7.

Plants were grown in 10.5 L pots arranged in a randomized complete block design, with three replications staggered in time. At the eighth leaf stage (approximately eight weeks old), plants were harvested. Three nodal roots from each plant were stored in 70% ethanol after cleaning. For observation, three root segments 5–10 cm from the root tip were stained with 0.5% toluidine blue in water and imaged at 40 × magnification (SMZ-U dissecting microscope, DS- Fil camera, Nikon, Tokyo, Japan). Root hair length and density were measured in separate images in ImageJ [94]. Length was measured from the edge of the root, and density was measured by counting the number of root hairs in an area of known dimensions [11].

### Association analysis

Broad sense heritability, or repeatability, was calculated as previously described [71] using the equation:

$${h}_{B}^{2}=\frac{{\upsigma }_{G}^{2}}{{\upsigma }_{G}^{2}+ \frac{{\upsigma }_{e}^{2}}{r}}$$

where σ2G represents genetic variance, σ2e represents residual variance, and r is the number of replicates. Genetic coefficients of variation (GCV) for each root trait were calculated as:

$$GCV(\mathrm{\%})=\frac{\sqrt{{\upsigma }_{G}^{2}}}{\upmu }*100$$

where μ is the population trait mean.

We analyzed association using five different methods, all using 314 RDP1 genotypes (referred to as *ALL*) (Supplemental Table 1).

First, we used the HDRA SNP set obtained from http://ricediversity.org/data/index.cfm. SNPs with minor allele frequency (MAF) > 0.05 in each subpopulation, as well as a count of at least three minor alleles and 30% maximum missing data, were included. When the analysis was run across all 314 lines in the panel, SNPs present at MAF > 0.05 in individual subpopulations were combined and three additional PC covariates were added to the model. When analyses were run on individual subpopulations (*indica*, *aus*, *temperate japonica* and *tropical japonica*), no covariates were used in the model. When analysis was run on the JAPONCIA clade, one covariate was used. Analysis was completed using the “RGWAS.normal” function of the RAINBOWR package with a genomic relationship matrix created using the A.mat method and mixed models residuals implemented through obtaining the zeta values through the “modify.data.res” function [73].

For our second analysis, we conducted LD-block genome-wide level analysis with the HDRA data. We created linkage disequilibrium (LD) blocks in PLINK (v 1.9) using the “—blocks” command and default values. These blocks were then used with the “RGWAS.multisnp” command from RAINBOWR with a Gaussian kernel, an additive test-effect, and a score-based test method.

The third, fourth, and fifth analyses used the RICE-RP SNP data, obtained from https://snp-seek.irri.org/download.zul (also available at https://www.ebi.ac.uk/ena/browser/view/PRJEB26328). For the third analysis, we created six, non-orthogonal smaller genome-wide datasets that were less computationally taxing and less likely to result in false-positive discoveries. SNPs were pruned in PLINK1.9 using both the indep and indep-pairwise functions using window sizes, step sizes, and variance inflation factor (VIF) thresholds or r2 thresholds (used by indep-pairwise) of 50, 5, 2 (VIF), 0.5 (r2); 50, 5, 1.5 (VIF), 0.2 (r2); 25, 5, 2 (VIF), 0.5 (r2). This resulted in six SNP sets, ranging in size from 705,712 to 1,189,943 SNPs. We then completed genome-wide association analysis using the “RGWAS.normal” function with the same MAF and missing parameters as the HDRA SNPs.

The fourth analysis used all RICE-RP SNPs from a single chromosome. Analysis was run using the same MAF and missingness parameters as whole-genome analysis.

Finally, for the fifth analysis we analyzed association on a gene-block level [73]. SNPs within gene coding regions according to the MSU genome v7 were combined into blocks along with SNPs in the putative promoter region (2 kb upstream) and 500 bp downstream and used for association analysis. We used the “RGWAS.multisnp” function with SNPs from the RAINBOWR package for this chromosome-wide analysis.

### Haplotype analysis

Genomic regions associated with root hair phenotypes were identified as regions with three or more SNPs within 200 kb of each other and significant below the *p* < 0.0001 threshold [95]. Regions were considered for further study if they were found in multiple RICE-RP SNP sets and the HDRA set. Figures were constructed using ggplot2 [96], and additional statistical analysis was completed using the rstatix package in R (R version 4.2, [97]). Local LD was estimated as previously described [98] with a 90^th^ percentile cutoff implemented as the critical *r*^2^ value. Haplotypes were constructed by using the identity of each SNP in the Nipponbare reference genome. Blocks were examined visually and representative SNPs from each region were used to create the representative haplotypes presented here. Only SNPs with less than 10% of lines carrying a heterozygous or missed read were used, and lines with heterozygosity at the selected SNPs were excluded from further analysis.

### Comparison with known root hair genes

Lists of known root hair genes in rice were constructed from the literature [33, 54–59, 61–63, 65, 66, 75, 99–108] (a complete list of these genes can be found in Supplemental Table 5). Comparisons were made between the generated lists of genes in our genomic regions and these genes. *Arabidopsis* homology was determined using the orthologous groups generated by the Rice Genome Annotation Project (http://rice.uga.edu/annotation_pseudo_apk.shtml).

### Cell length and hair distribution analysis

To determine if cell size was influencing root hair density, a selection of lines (Ming Hui, CO18, RTS4, SL22-613, Sigadis, China 1039, Dawebyan, and Short Grain) were grown in conical containers (2.7 × 10″, 656 mL, Deepot Cell, D20T, Stewe and Sons, Tangent, OR, USA) until the third leaf stage (approximately three weeks of growth). The medium consisted of 40% sand, 40% vermiculite, 15% soil (Hagerstown-Opequon, fine-clayey, mixed, mesic (Type Hapludalf)), and 5% perlite. Plants were fertigated with Yoshida nutrient solution and grown in the same greenhouses as previously described.

At harvest, three nodal roots were collected from each plant and stored in 70% ethanol. A segment 5–10 cm from the root tip was isolated from each root and stained with 0.05% toluidine blue. A collection of z-stack images (minimum eight images per sample) was collected on a Nikon Diaphot at 200 × equipped with a digital camera (NIKON DS-Fi1, Nikon, Tokyo, Japan). Three roots per plant were imaged from four plants per genotype. As many cells as could be detected from the center of the root (i.e., within the focal plane) were measured and designated as hair and non-hair cells. Image stacks were reconstructed in ImageJ [94] and hair and non-hair cells were identified and outlined to measure cell length and width.

### *cis*-acting root hair element sequence detection

We queried our generated list of candidate genes for *cis*-acting elements in the putative promoters (2 kb upstream from the transcription start site) known as root hair elements (RHE) by downloading FASTA promoter sequences from the Rice Genome Hub (https://shinyapps.southgreen.fr/app/downloadgenesequences). We queried the 16 and 17 bp long sequences previously identified as RHEs using the Find Individual Motif Occurrences (FIMO) tool of the MEME suite (http://meme-suite.org/tools/fimo) [109]. The sequence used was [WHHDTGNNN(N)KCACGWH], where W = A/T, H = A/T/C, D = G/T/A, K = G/T, and N = A/T/C/G [110], which is slightly less strict than other RHE sequence queries [76].

## Supplementary Information

**Additional file 1: Supplemental Tables 1-7. Supplemental Table 1.** Genotypes and mean phenotypes used in this study. **Supplemental Table 2:** broad-sense heritability (h2) and genotypic coefficient of variance by subpopulation for RHL and RHD. **Supplemental Table 3:** Genes significantly associated with RHD and RHL using gene-block chromosome-wide analysis. **Supplemental Table 4:** All genes within significant genomic regions as identified by GWAS. **Supplemental Table 5:** Genes previously identified to play a role in rice RH formation or growth. **Supplemental Table 6:** Genes with RHE (cis-acting root hair elements) in the putative promoter region. **Supplemental Table 7:** Condensed list of candidate genes.

**Additional file 2: Supplemental Figures 1-32. Supplemental Figure 1.** Genome- wide association analysis using the HDRA SNPs, HDRA LD blocks, or pruned sets of the RICE-RP SNPs (p1-p6) for *ALL* RHD. **Supplemental Figure 2.** *ALL *RHD chromosome-wide association analysis using the RICE-RP SNPs or the SNPs collapsed into gene blocks. **Supplemental Figure 3.** Genome-wide association analysis using the HDRA SNPs, HDRA LD blocks, or pruned sets of the RICE-RP SNPs (p1-p6) for *ALL *RHL. **Supplemental Figure 4.** *ALL *RHL chromosome-wide association analysis using the RICE-RP SNPs or the SNPs collapsed into gene blocks. **Supplemental Figure 5.** Genome-wide association analysis using the HDRA SNPs, HDRA LD blocks, or pruned sets of the RICE-RP SNPs (p1-p6) for *aus *RHD. **Supplemental Figure 6.** *aus *RHD chromosome-wide association analysis using the RICE-RP SNPs or the SNPs collapsed into gene blocks. **Supplemental Figure 7.** Genome-wide association analysis using the HDRA SNPs, HDRA LD blocks, or pruned sets of the RICE-RP SNPs (p1-p6) for *aus *RHL. **Supplemental Figure 8.** *aus *RHL chromosome-wide association analysis using the RICE-RP SNPs or the SNPs collapsed into gene blocks. **Supplemental Figure 9.** Genome-wide association analysis using the HDRA SNPs, HDRA LD blocks, or pruned sets of the RICE-RP SNPs (p1-p6) for *ind *RHD. **Supplemental Figure 10.** *ind *RHD chromosome-wide association analysis using the RICE-RP SNPs or the SNPs collapsed into gen blocks. **Supplemental Figure 11.** Genome-wide association analysis using the HDRA SNPs, HDRA LD blocks, or pruned sets of the RICE-RP SNPs (p1-p6) for *ind *RHL. **Supplemental Figure 12.** *ind *RHL chromosome-wide association analysis using the RICE-RP SNPs or the SNPs collapsed into gene blocks. **Supplemental Figure 13.** Genome-wide association analysis using the HDRA SNPs, HDRA LD blocks, or pruned sets of the RICE-RP SNPs (p1-p6) for *JAPONICA *RHD. **Supplemental Figure 14.** *JAPONICA *RHD chromosome-wide association analysis using the RICE-RP SNPs or the SNPs collapsed into gene blocks. **Supplemental Figure 15.** Genome-wide association analysis using the HDRA SNPs, HDRA LD blocks, or pruned sets of the RICE-RP SNPs (p1-p6) for *JAPONICA *RHL. **Supplemental Figure 16.** *JAPONICA *RHL chromosome-wide association analysis using the RICE-RP SNPs or the SNPs collapsed into gene blocks. **Supplemental Figure 17.** Genome-wide association analysis using the HDRA SNPs, HDRA LD blocks, or pruned sets of the RICE-RP SNPs (p1-p6) for *tej *RHD. **Supplemental Figure 18.** *tej *RHD chromosome-wide association analysis using the RICE-RP SNPs or the SNPs collapsed into gene blocks. **Supplemental Figure 19.** Genome-wide association analysis using the HDRA SNPs, HDRA LD blocks, or pruned sets of the RICE-RP SNPs (p1-p6) for *tej *RHL. **Supplemental Figure 20.** *tej *RHL chromosome-wide association analysis using the RICE-RP SNPs or the SNPs collapsed into gene blocks. **Supplemental Figure 21.** Genome-wide association analysis using the HDRA SNPs, HDRA LD blocks, or pruned sets of the RICE-RP SNPs (p1-p6) for *trj *RHD. **Supplemental Figure 22.** *trj *RHD chromosome-wide association analysis using the RICE-RP SNPs or the SNPs collapsed into gene blocks. **Supplemental Figure 23.** Genome-wide association analysis using the HDRA SNPs, HDRA LD blocks, or pruned sets of the RICE-RP SNPs (p1-p6) for *trj *RHD. **Supplemental Figure 24.** *trj *RHL chromosome-wide association analysis using the RICE-RP SNPs or the SNPs collapsed into gene blocks. **Supplemental Figure 25.** Haplotype exploration of the region on chromosome 5 associated with RHL in *ALL. ***Supplemental Figure 26.** Region D4 from *ind *for RHD on chromosome 5 lacks support across multiple association runs. **Supplemental Figure 27.** Region D7 associated with RHD from *ALL *and *JAPONICA *lines. **Supplemental Figure 28.** Region D9 associated with RHD in *trj* on chromosome 8. **Supplemental Figure 29.** Region D10 associated with RHD in *ALL* on chromosome 9. **Supplemental Figure 30.** Haplotype exploration of the region on chromosome 1 associated with RHD in *aus*. **Supplemental Figure 31.** Haplotype exploration of the region on chromosome 10 associated with RHD in *ALL* and *JAPONICA*. **Supplemental Figure 32.** Haplotype exploration of region D14 on chromosome 12 associated with RHD in *ALL* and *JAPONICA*.

## Data Availability

All data generated or analyzed during this study are included in this published article and its supplementary information files. RDP1 lines are available from the USDA-ARS, Dale Bumpers National Rice Research Center, Stuttgart, Arkansas, USA, Genetic Stocks *Oryza* Collection. The HDRA SNP data has been deposited in the NCBI dbSNP database (batch ID 1062024) and the GEO database (accession ID: GSE71553) and is also available at http://ricediversity.org/data/index.cfm. RICE RP SNP data is available at https://snp-seek.irri.org/download.zul and also at https://www.ebi.ac.uk/ena/browser/view/PRJEB26328.

## Abbreviations

ALL: Entire RDP-1 population
FIMO: Find Individual Motif Occurrences
GW: Genome-wide
GWA: Genome-wide association
HDRA: High density rice array
ind: (Lower case) subpopulation indica
LD: Linkage disequilibrium
MAF: Minor allele frequency
MS-SNP: Most significant SNP
QTL: Quantitative trait locus
RDP-1: Rice diversity panel-1
RHE: Root hair elements
RICE-RP: Rice reference panel
RHD: Root hair density
RHL: Root hair length
SNP: Single nucleotide polymorphism
tej: Subpopulation temperate japonica
trj: Subpopulation tropical japonica
VIF: Variance inflation factor
