# Supplementary material for: Genome wide association analysis of root hair traits in rice reveals novel genomic regions controlling epidermal cell differentiation
Source: BMC Plant Biol. 2023 Jan 4;23:6. doi: 10.1186/s12870-022-04026-5 (PMC9811729; doi:10.1186/s12870-022-04026-5)
Supplement: Supplementary file 2 — Additional file 2: Supplemental Figures 1-32. Supplemental Figure 1. Genome- wide association analysis using the HDRA SNPs, HDRA LD blocks, or pruned sets of the RICE-RP SNPs (p1-p6) for ALL RHD. Supplemental Figure 2. ALL RHD chromosome-wide association analysis using the RICE-RP SNPs or the SNPs collapsed into gene blocks. Supplemental Figure 3. Genome-wide association analysis using the HDRA SNPs, HDRA LD blocks, or pruned sets of the RICE-RP SNPs (p1-p6) for ALL RHL. Supplemental Figure 4. ALL RHL chromosome-wide association analysis using the RICE-RP SNPs or the SNPs collapsed into gene blocks. Supplemental Figure 5. Genome-wide association analysis using the HDRA SNPs, HDRA LD blocks, or pruned sets of the RICE-RP SNPs (p1-p6) for aus RHD. Supplemental Figure 6. aus RHD chromosome-wide association analysis using the RICE-RP SNPs or the SNPs collapsed into gene blocks. Supplemental Figure 7. Genome-wide association analysis using the HDRA SNPs, HDRA LD blocks, or pruned sets of the RICE-RP SNPs (p1-p6) for aus RHL. Supplemental Figure 8. aus RHL chromosome-wide association analysis using the RICE-RP SNPs or the SNPs collapsed into gene blocks. Supplemental Figure 9. Genome-wide association analysis using the HDRA SNPs, HDRA LD blocks, or pruned sets of the RICE-RP SNPs (p1-p6) for ind RHD. Supplemental Figure 10. ind RHD chromosome-wide association analysis using the RICE-RP SNPs or the SNPs collapsed into gen blocks. Supplemental Figure 11. Genome-wide association analysis using the HDRA SNPs, HDRA LD blocks, or pruned sets of the RICE-RP SNPs (p1-p6) for ind RHL. Supplemental Figure 12. ind RHL chromosome-wide association analysis using the RICE-RP SNPs or the SNPs collapsed into gene blocks. Supplemental Figure 13. Genome-wide association analysis using the HDRA SNPs, HDRA LD blocks, or pruned sets of the RICE-RP SNPs (p1-p6) for JAPONICA RHD. Supplemental Figure 14. JAPONICA RHD chromosome-wide association analysis using the RICE-RP SNPs or the SNPs collaps [file 12870_2022_4026_MOESM2_ESM.pdf]

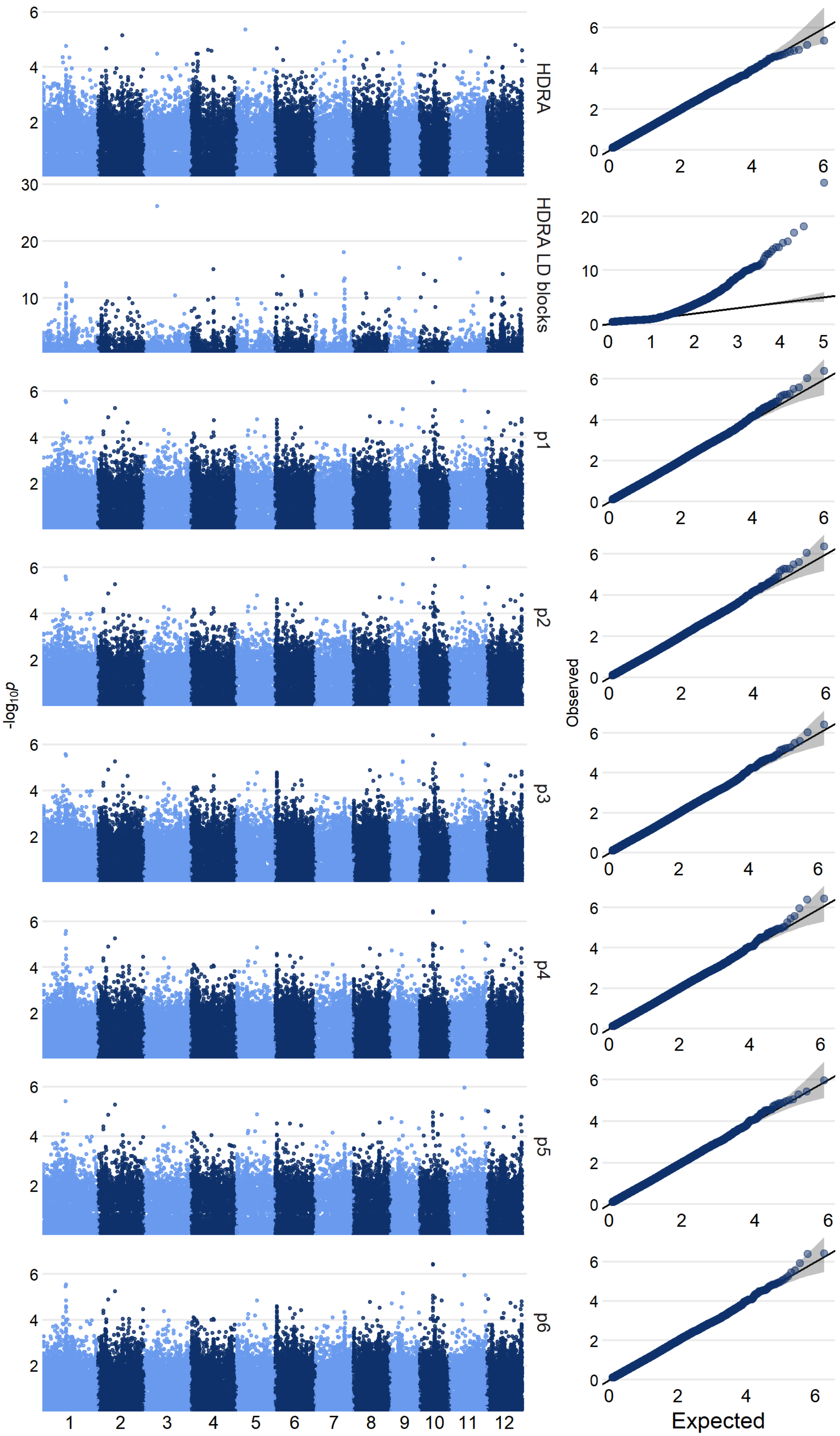

Supplemental Figure 1. Genome-wide association analysis using the HDRA SNPs, HDRA LD blocks, or pruned sets of the RICE-RP SNPs (p1-p6) for *ALL* RHD. Manhattan plots are in the left column and their corresponding QQ plots are in the right column.

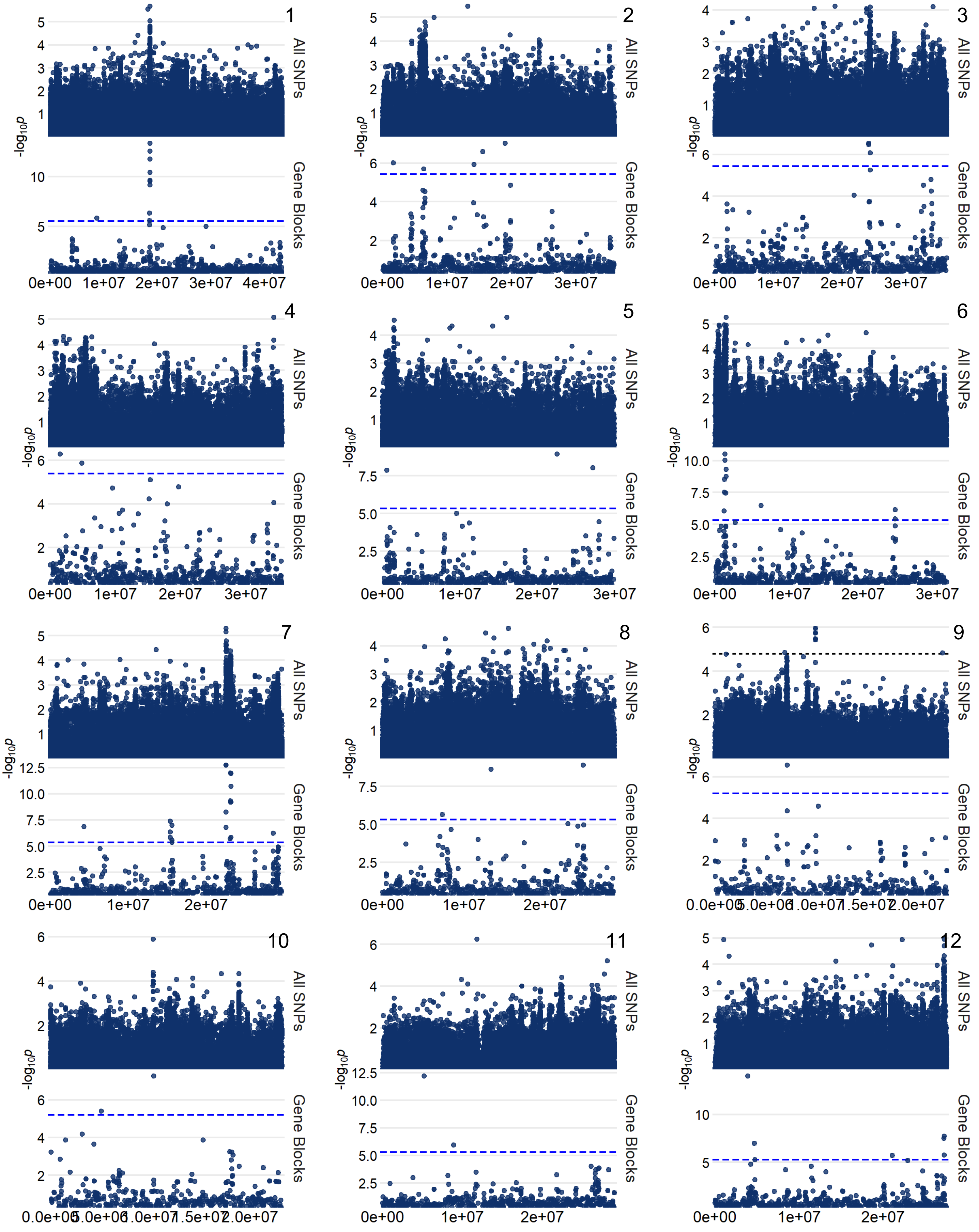

Supplemental Figure 2 *ALL* RHD Chromosome-wide association analysis using the RICE-RP SNPs (upper) or the SNPs collapsed into gene blocks (lower) for chromosomes 1-12, indicated in the upper right. Blue lines indicate significance at the Bonferroni adjusted p-value < 0.05 for individual SNPs and 0.01 for gene blocks.

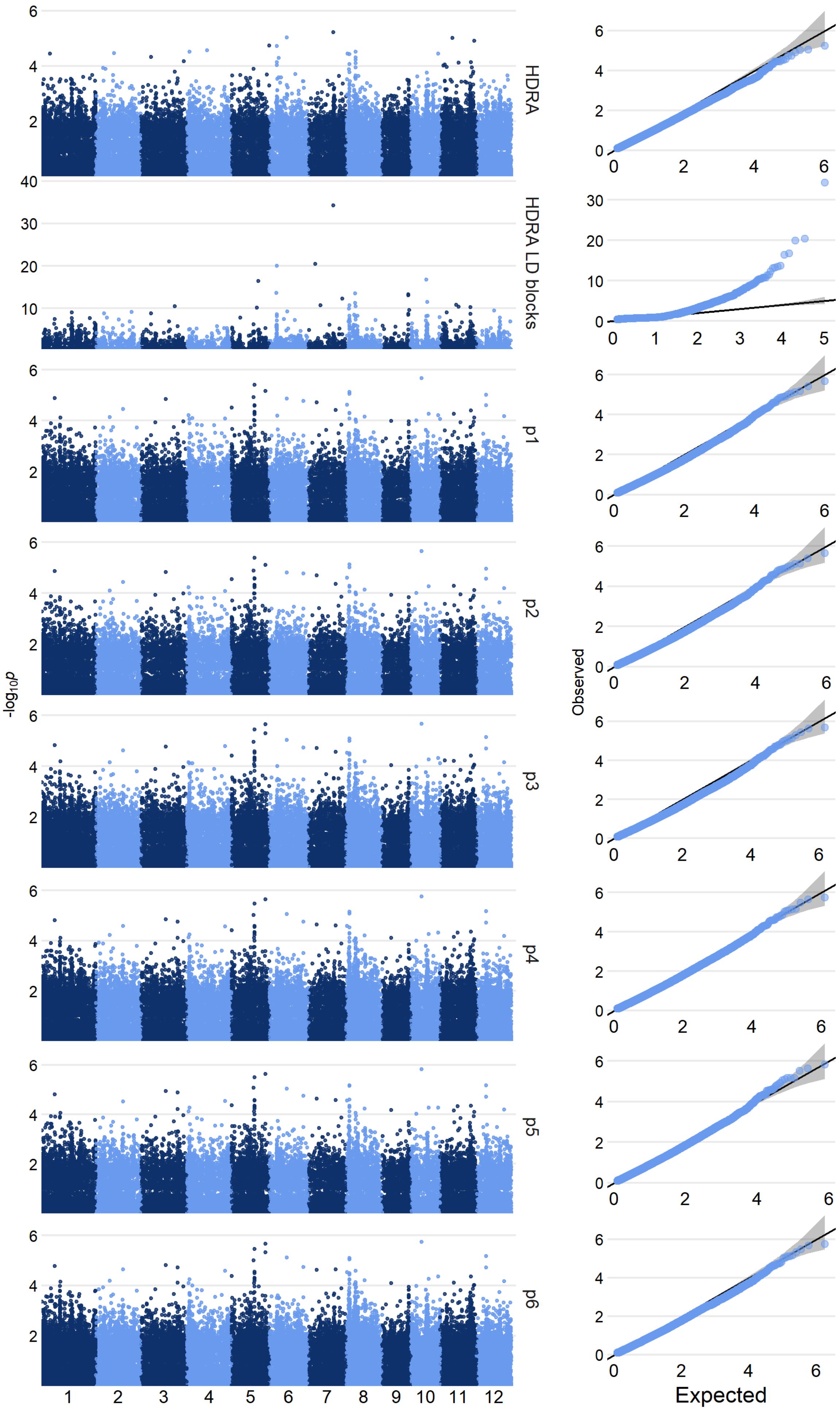

Supplemental Figure 3. Genome-wide association analysis using the HDRA SNPs, HDRA LD blocks, or pruned sets of the RICE-RP SNPs (p1-p6) for *ALL* RHL. Manhattan plots are in the left column and their corresponding QQ plots are in the right column.

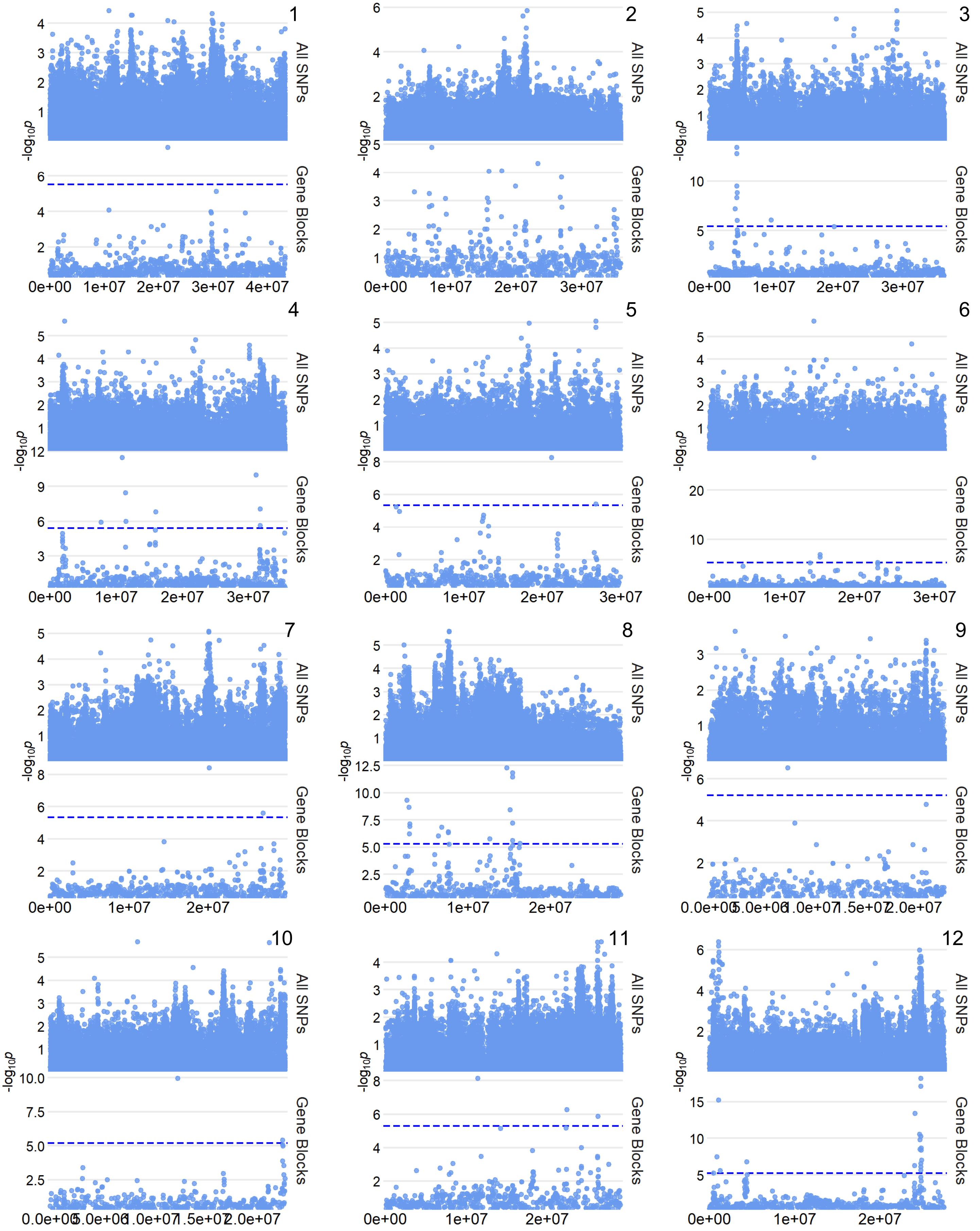

Supplemental Figure 4 *ALL* RHL CW Chromosome-wide association analysis using the RICE-RP SNPs (upper) or the SNPs collapsed into gene blocks (lower) for chromosomes 1-12, indicated in the upper right. Blue lines indicate significance at the Bonferroni adjusted p-value < 0.05 for individual SNPs and 0.01 for gene blocks.

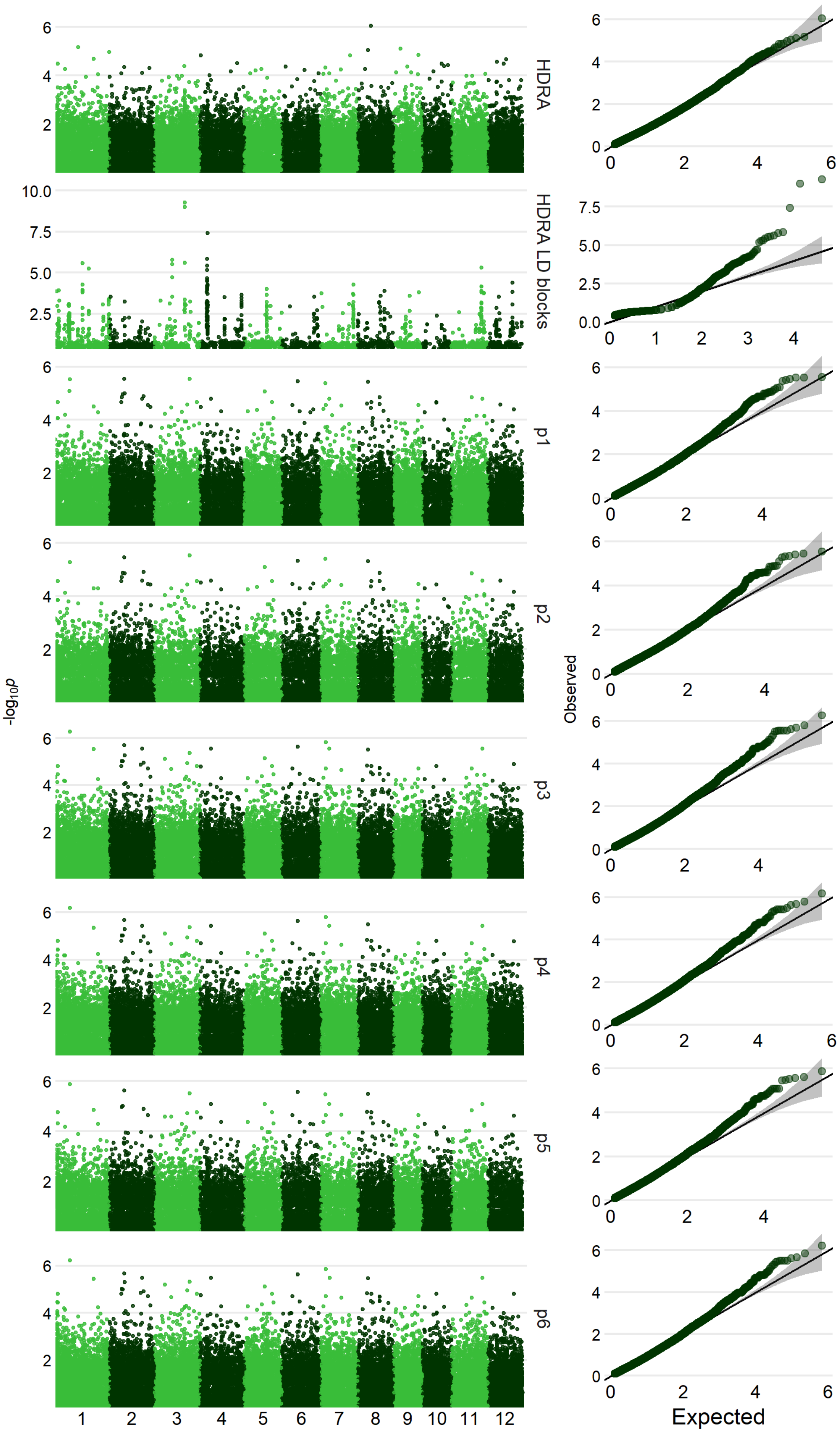

Supplemental Figure 5. Genome-wide association analysis using the HDRA SNPs, HDRA LD blocks, or pruned sets of the RICE-RP SNPs (p1-p6) for *aus* RHD. Manhattan plots are in the left column and their corresponding QQ plots are in the right column.

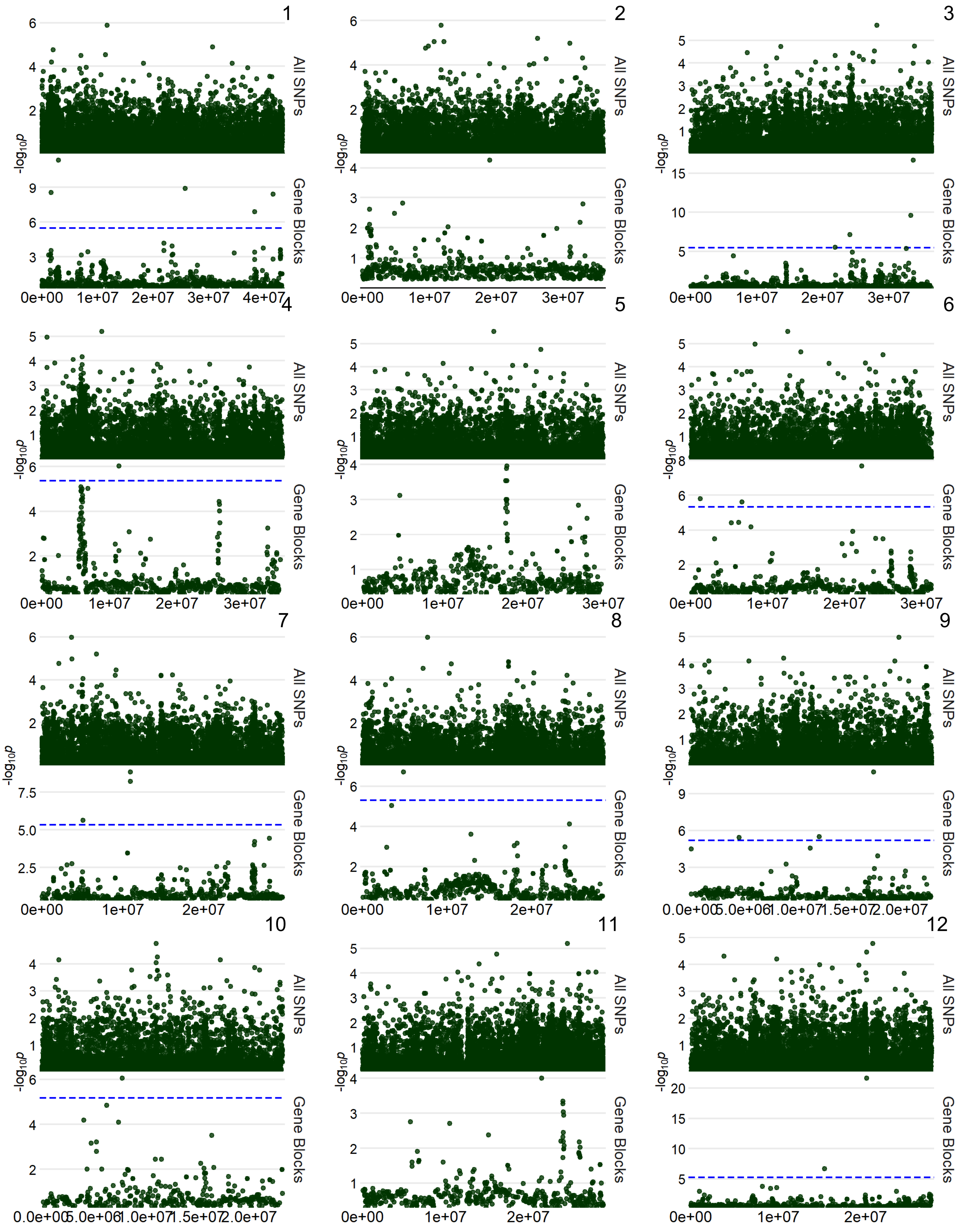

Supplemental Figure 6 *aus* RHD Chromosome-wide association analysis using the RICE-RP SNPs (upper) or the SNPs collapsed into gene blocks (lower) for chromosomes 1-12, indicated in the upper right. Blue lines indicate significance at the Bonferroni adjusted p-value < 0.05 for individual SNPs and 0.01 for gene blocks.

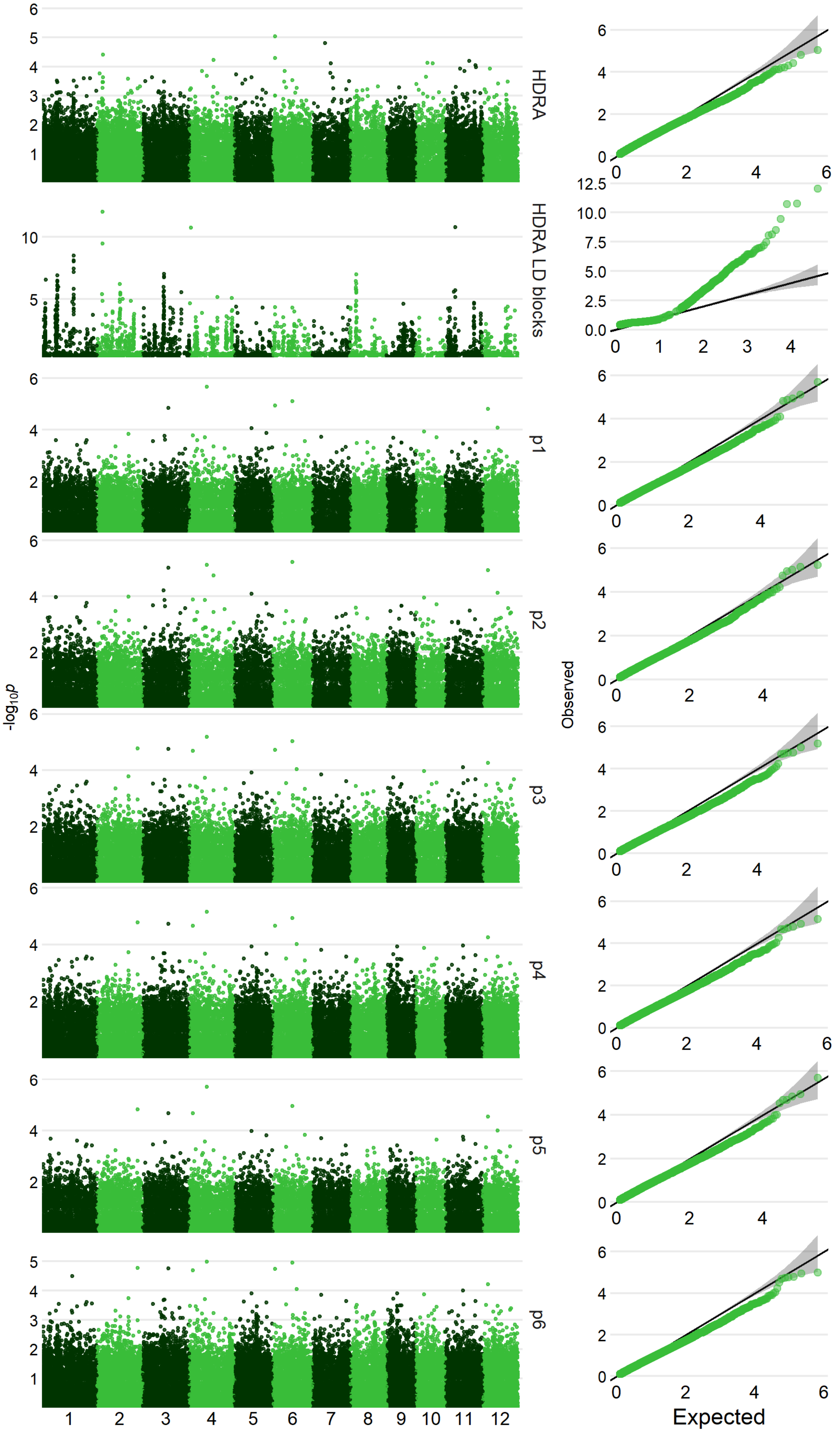

Supplemental Figure 7. Genome-wide association analysis using the HDRA SNPs, HDRA LD blocks, or pruned sets of the RICE-RP SNPs (p1-p6) for *aus* RHL. Manhattan plots are in the left column and their corresponding QQ plots are in the right column.

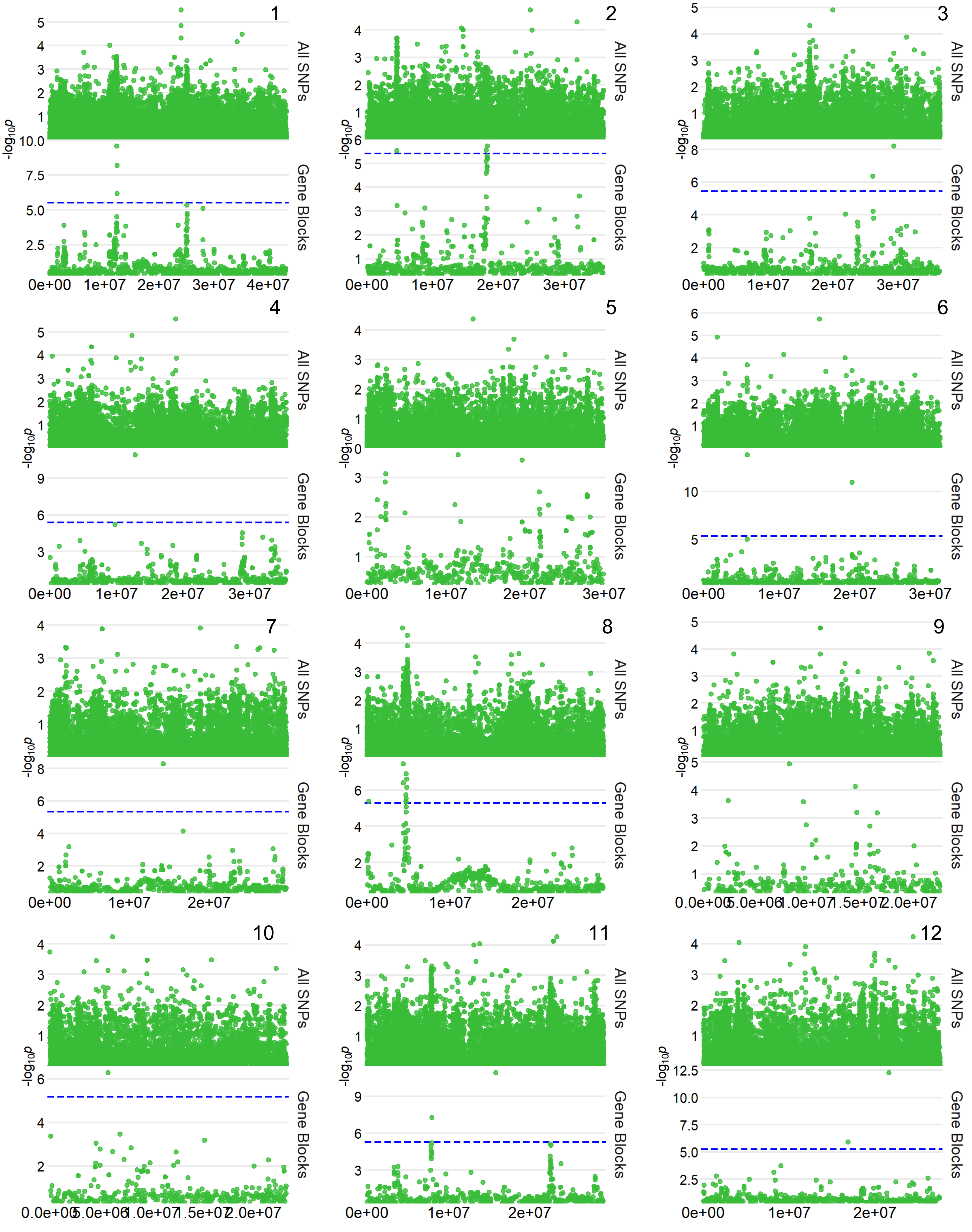

Supplemental Figure 8 *aus* RHL Chromosome-wide association analysis using the RICE-RP SNPs (upper) or the SNPs collapsed into gene blocks (lower) for chromosomes 1-12, indicated in the upper right. Blue lines indicate significance at the Bonferroni adjusted p-value < 0.05 for individual SNPs and 0.01 for gene blocks.

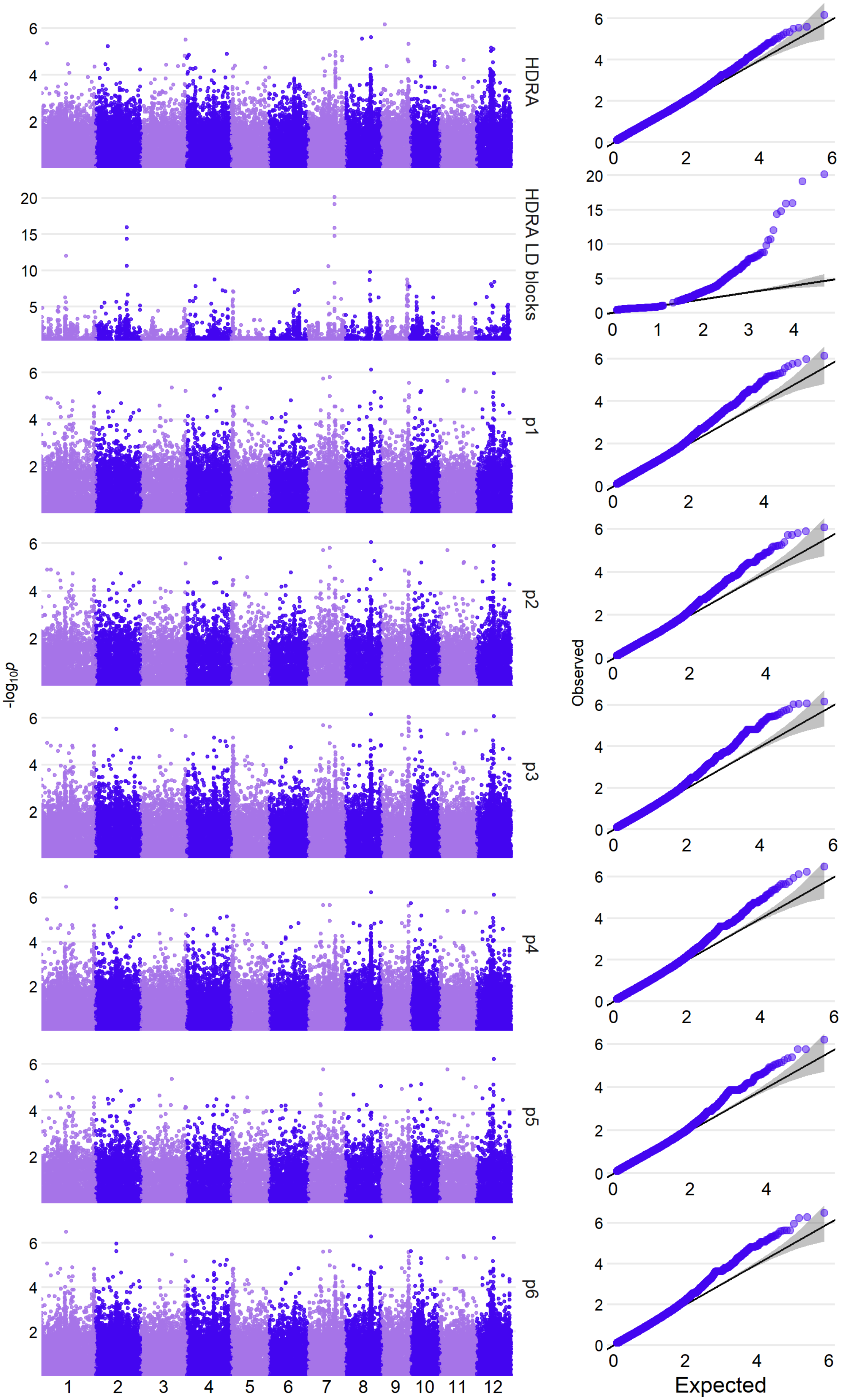

Supplemental Figure 9. Genome-wide association analysis using the HDRA SNPs, HDRA LD blocks, or pruned sets of the RICE-RP SNPs (p1-p6) for *ind* RHD. Manhattan plots are in the left column and their corresponding QQ plots are in the right column.

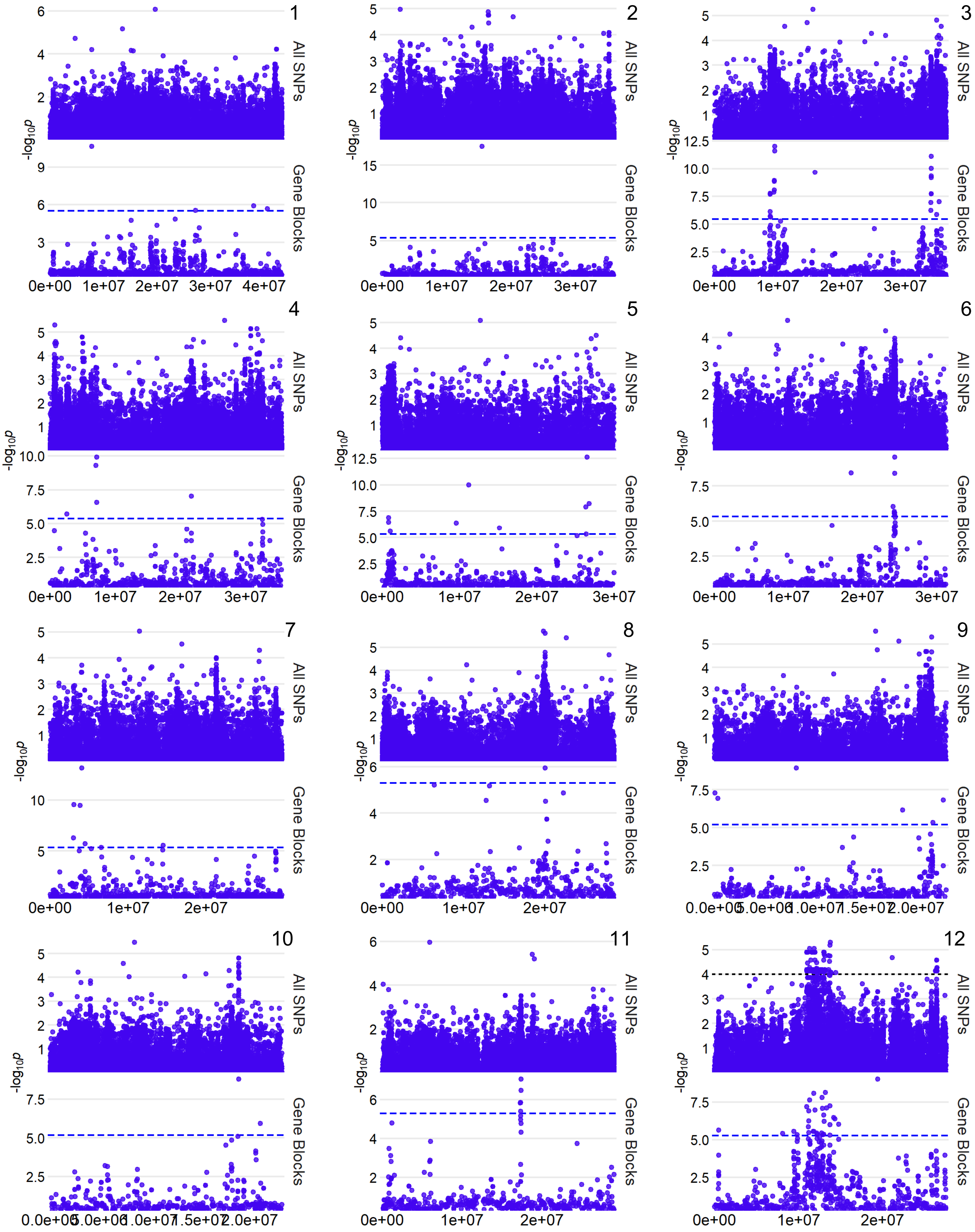

Supplemental Figure 10 *ind* RHD Chromosome-wide association analysis using the RICE-RP SNPs (upper) or the SNPs collapsed into gene blocks (lower) for chromosomes 1-12, indicated in the upper right. Blue lines indicate significance at the Bonferroni adjusted p-value < 0.05 for individual SNPs and 0.01 for gene blocks.

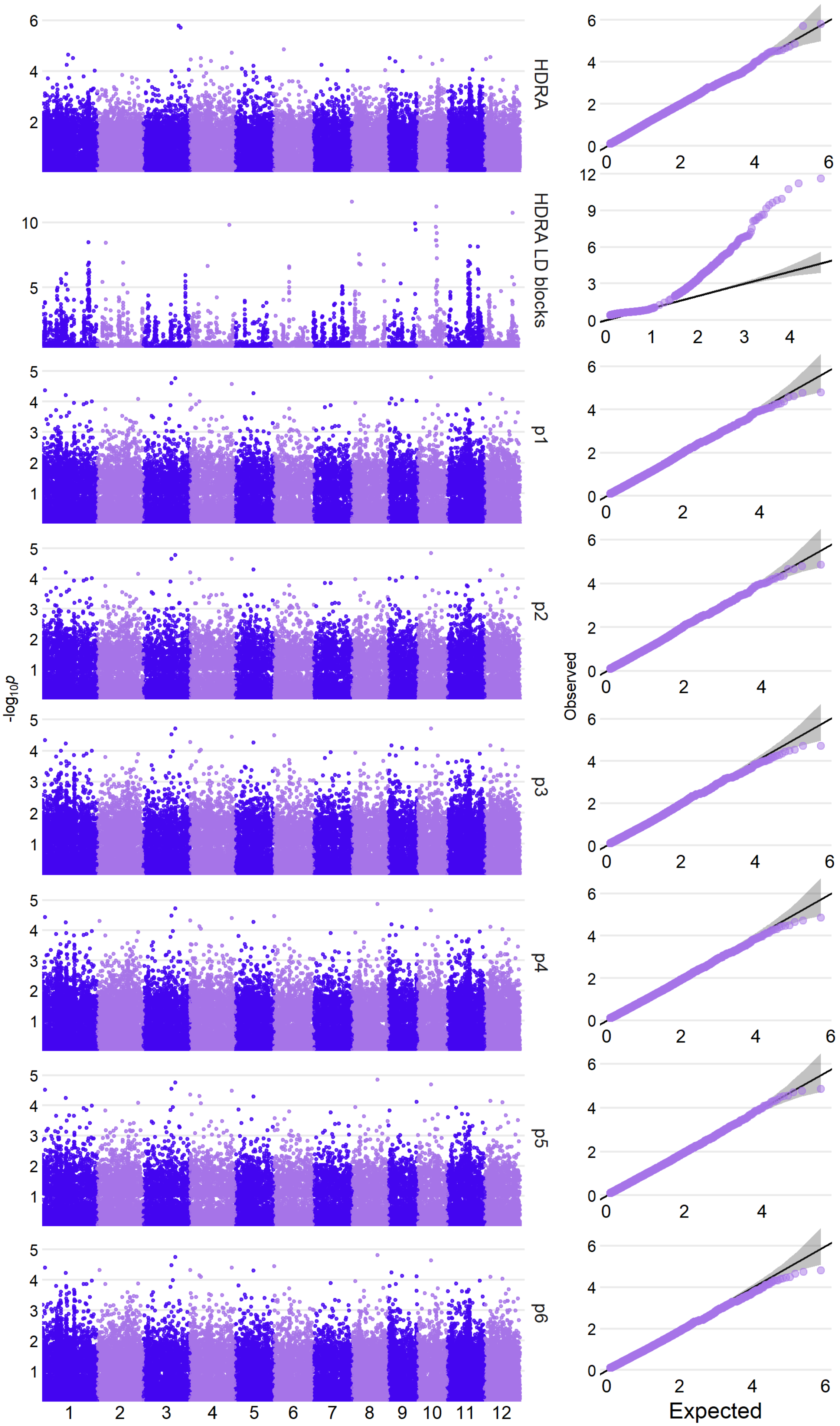

Supplemental Figure 11. Genome-wide association analysis using the HDRA SNPs, HDRA LD blocks, or pruned sets of the RICE-RP SNPs (p1-p6) for *ind* RHL. Manhattan plots are in the left column and their corresponding QQ plots are in the right column.

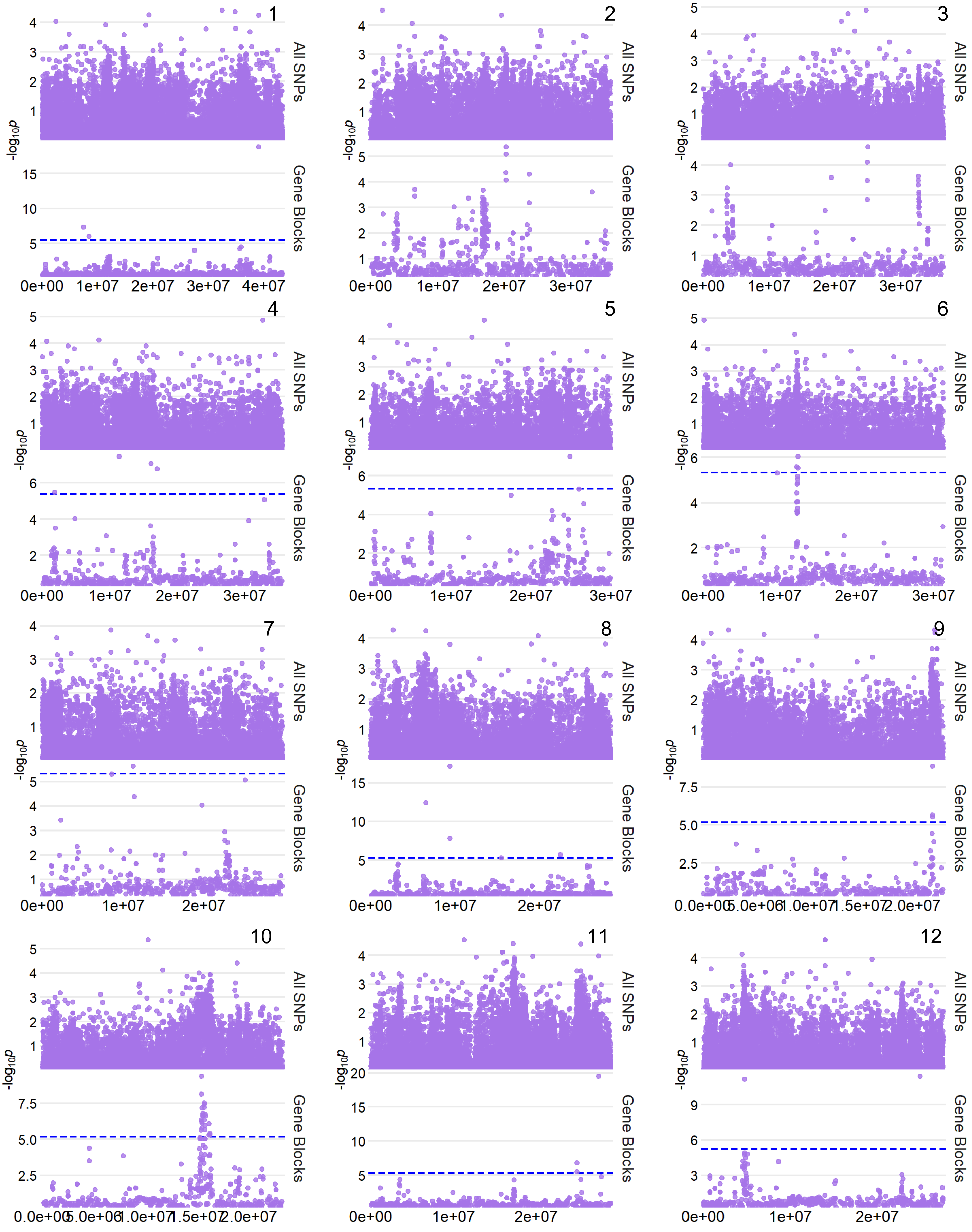

Supplemental Figure 12 *ind* RHL Chromosome-wide association analysis using the RICE-RP SNPs (upper) or the SNPs collapsed into gene blocks (lower) for chromosomes 1-12, indicated in the upper right. Blue lines indicate significance at the Bonferroni adjusted p-value < 0.05 for individual SNPs and 0.01 for gene blocks.

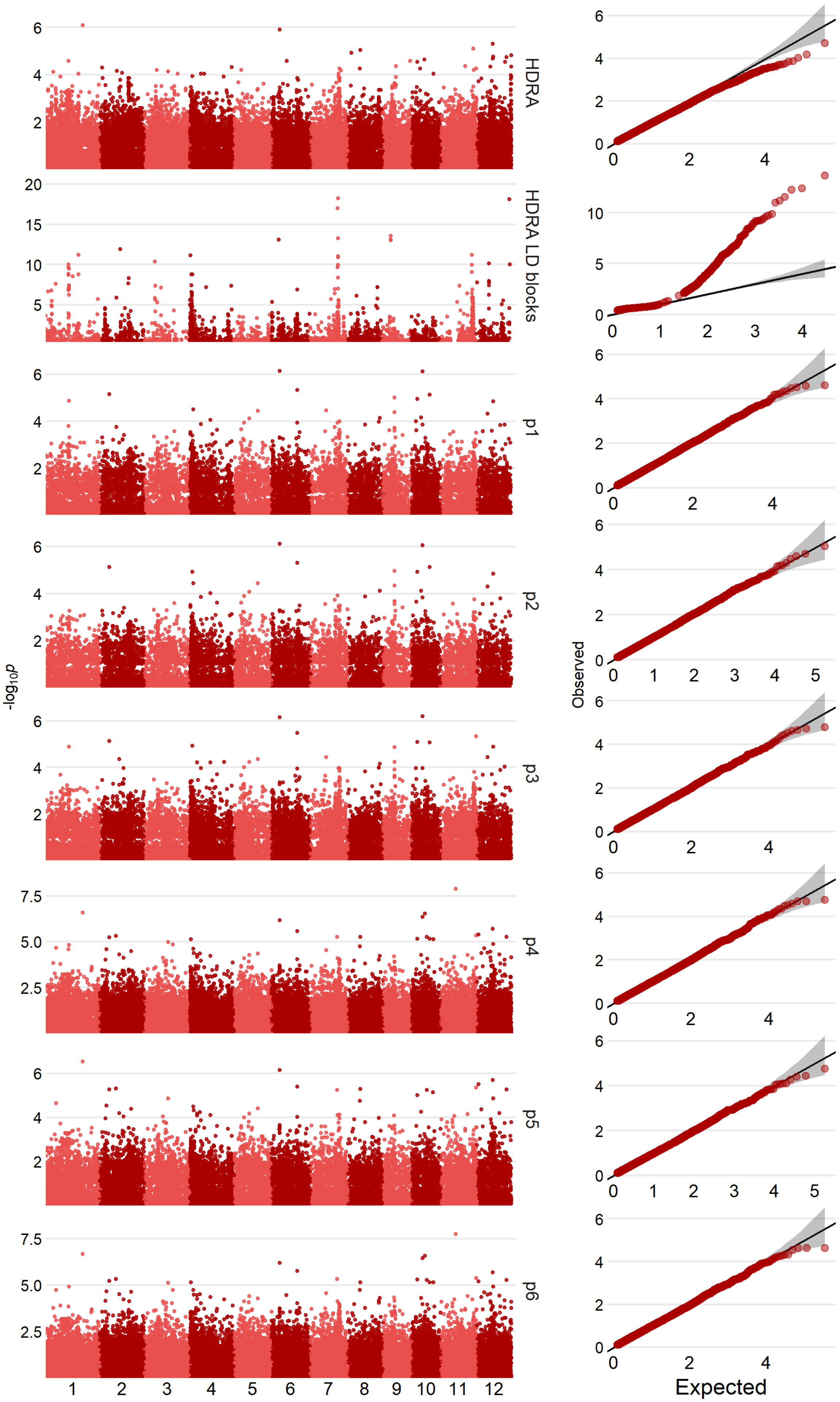

Supplemental Figure 13. Genome-wide association analysis using the HDRA SNPs, HDRA LD blocks, or pruned sets of the RICE-RP SNPs (p1-p6) for *JAPONICA* RHD. Manhattan plots are in the left column and their corresponding QQ plots are in the right column.

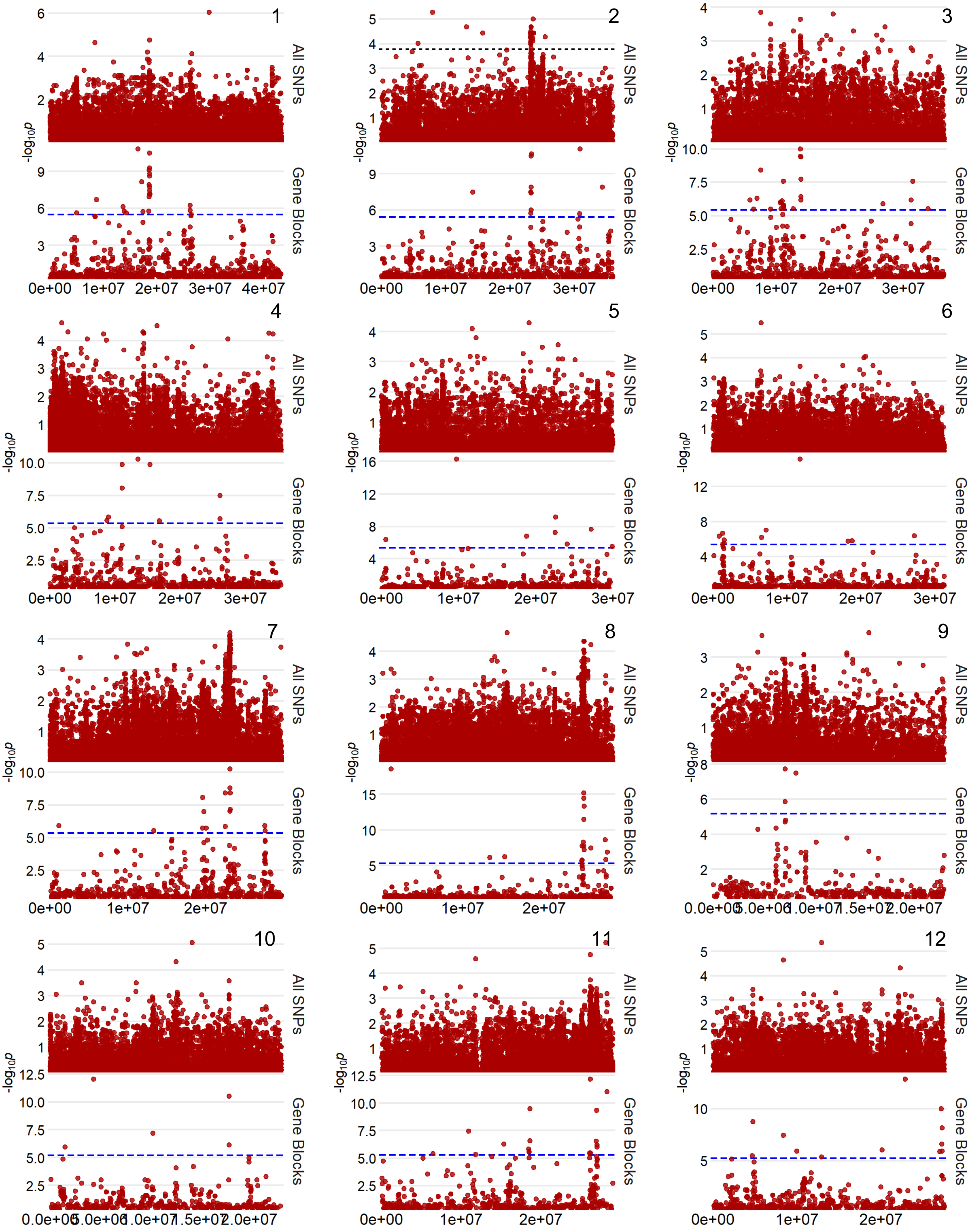

Supplemental Figure 14 *JAPONICA* RHD Chromosome-wide association analysis using the RICE-RP SNPs (upper) or the SNPs collapsed into gene blocks (lower) for chromosomes 1-12, indicated in the upper right. Blue lines indicate significance at the Bonferroni adjusted p-value < 0.05 for individual SNPs and 0.01 for gene blocks.

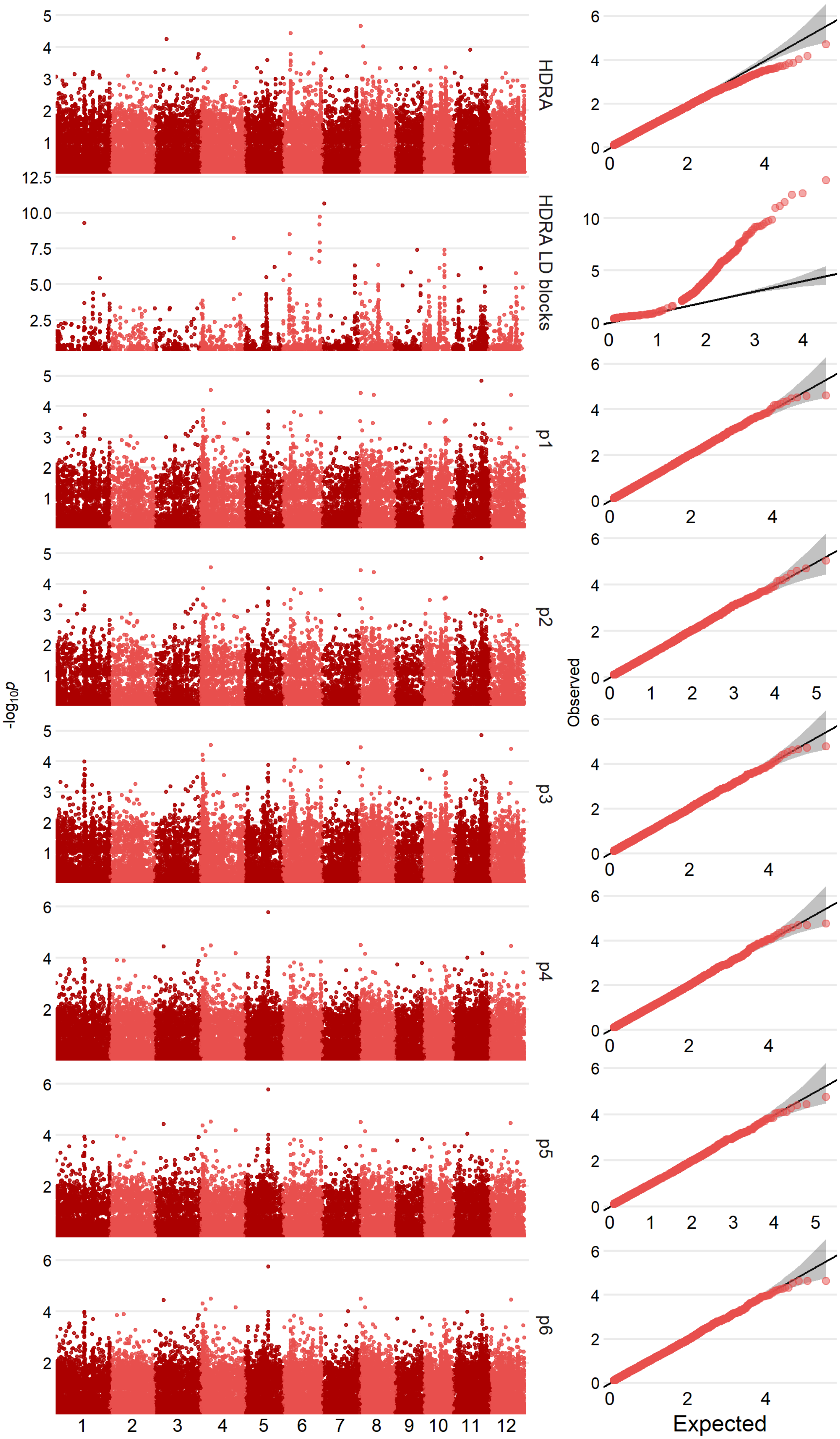

Supplemental Figure 15. Genome-wide association analysis using the HDRA SNPs, HDRA LD blocks, or pruned sets of the RICE-RP SNPs (p1-p6) for *JAPONICA* RHL. Manhattan plots are in the left column and their corresponding QQ plots are in the right column.

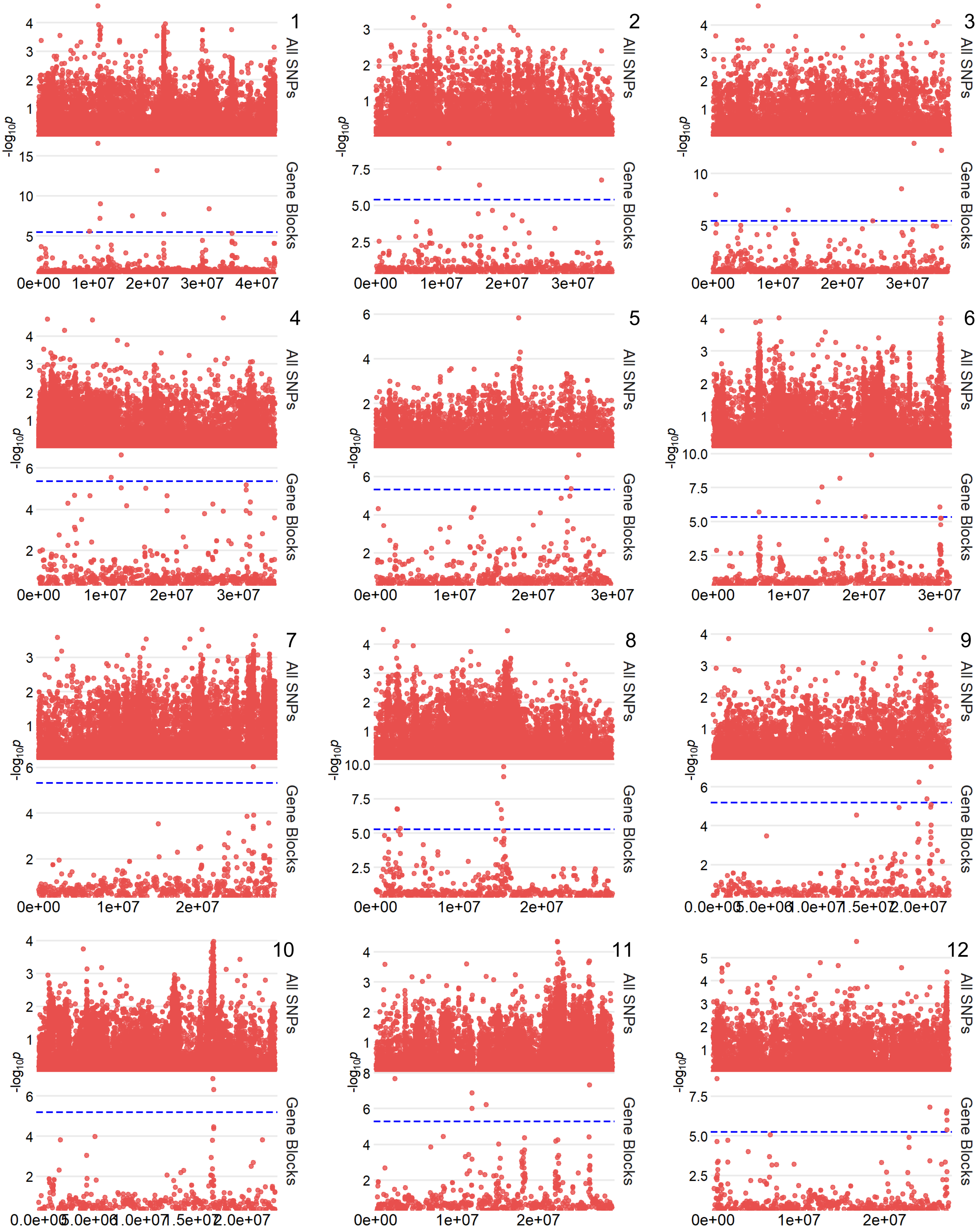

Supplemental Figure 16 *JAPONICA* RHL Chromosome-wide association analysis using the RICE-RP SNPs (upper) or the SNPs collapsed into gene blocks (lower) for chromosomes 1-12, indicated in the upper right. Blue lines indicate significance at the Bonferroni adjusted p-value < 0.05 for individual SNPs and 0.01 for gene blocks.

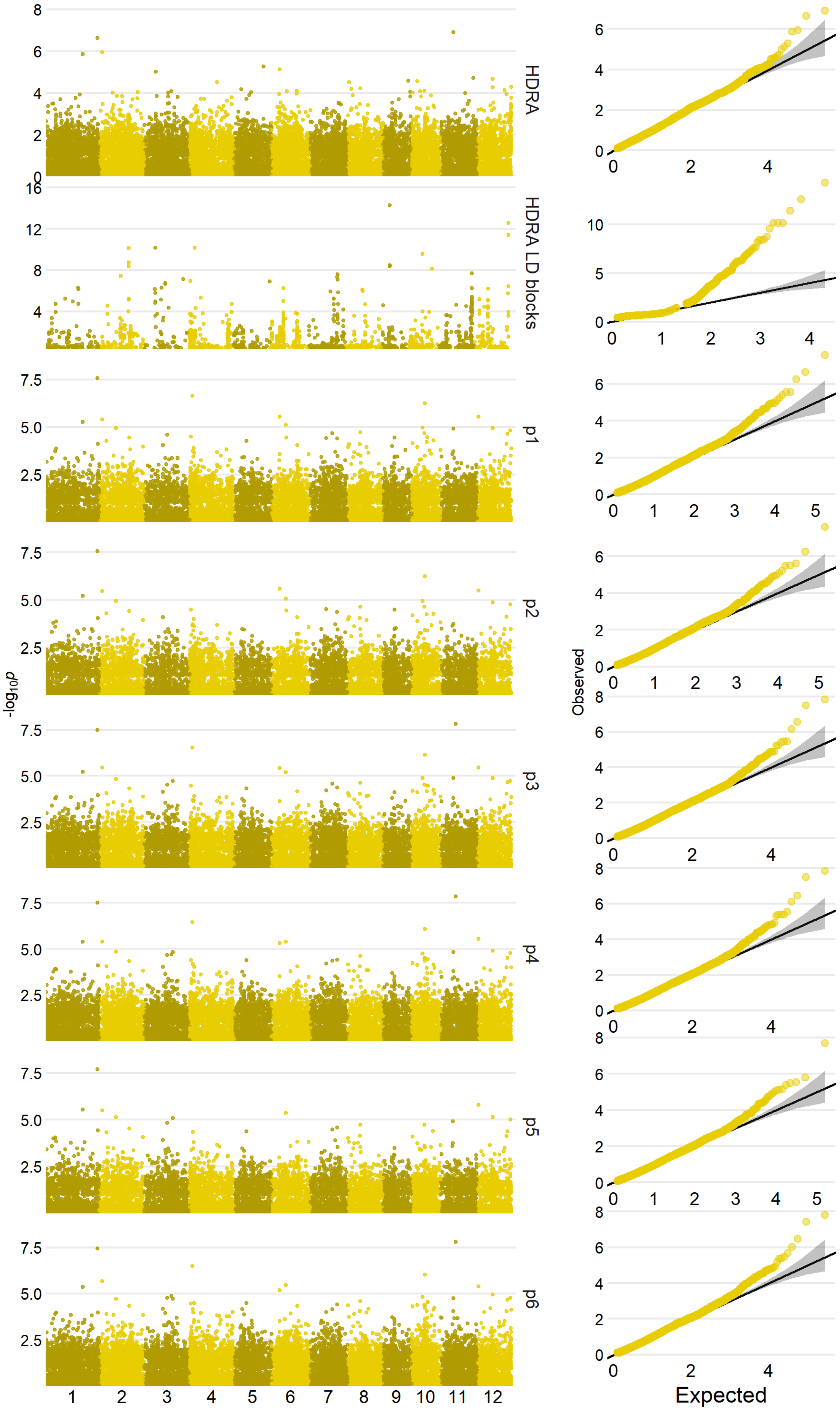

Supplemental Figure 17. Genome-wide association analysis using the HDRA SNPs, HDRA LD blocks, or pruned sets of the RICE-RP SNPs (p1-p6) for *tej* RHD. Manhattan plots are in the left column and their corresponding QQ plots are in the right column.

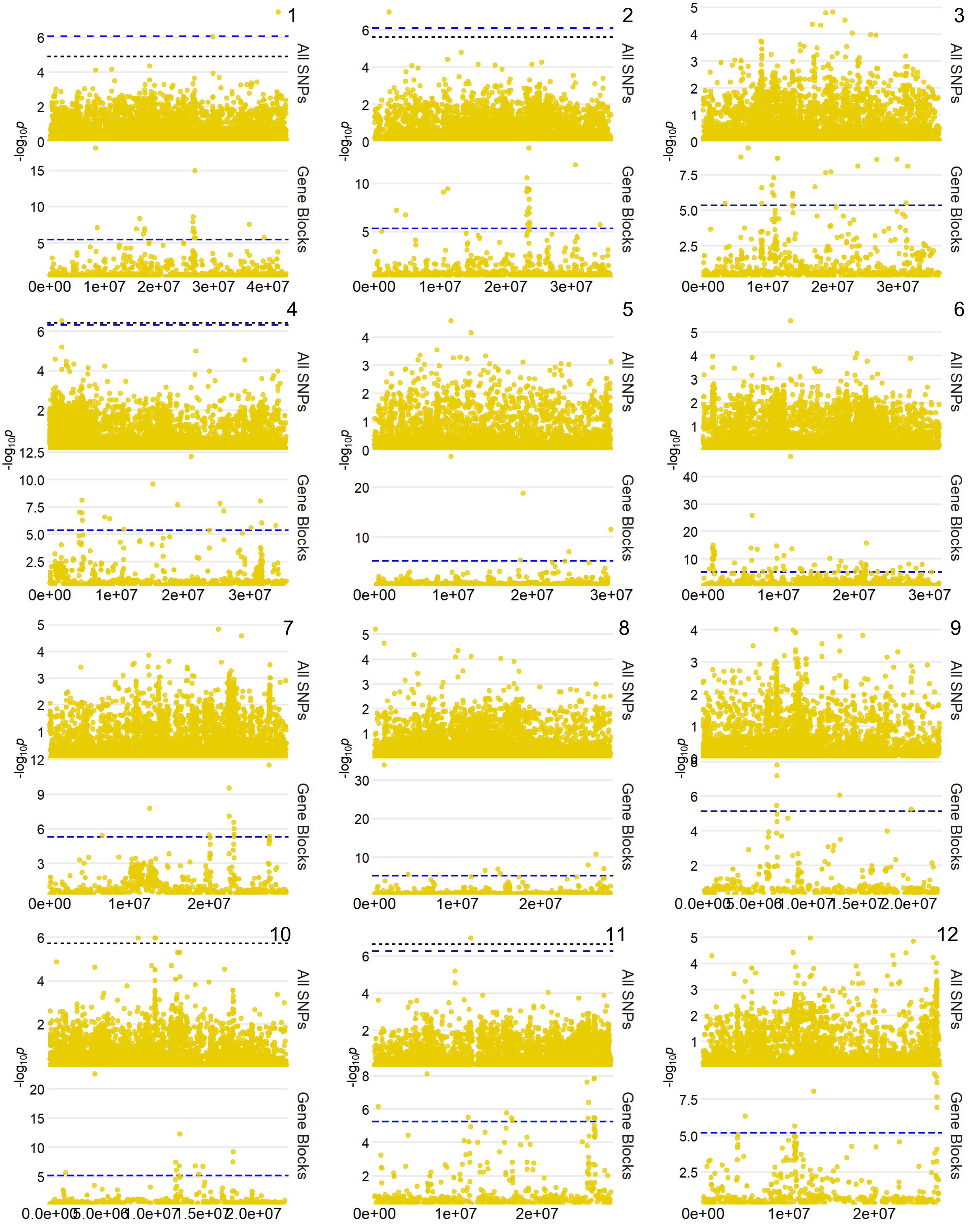

Supplemental Figure 18 *tej* RHD Chromosome-wide association analysis using the RICE-RP SNPs (upper) or the SNPs collapsed into gene blocks (lower) for chromosomes 1-12, indicated in the upper right. Blue lines indicate significance at the Bonferroni adjusted p-value < 0.05 for individual SNPs and 0.01 for gene blocks.

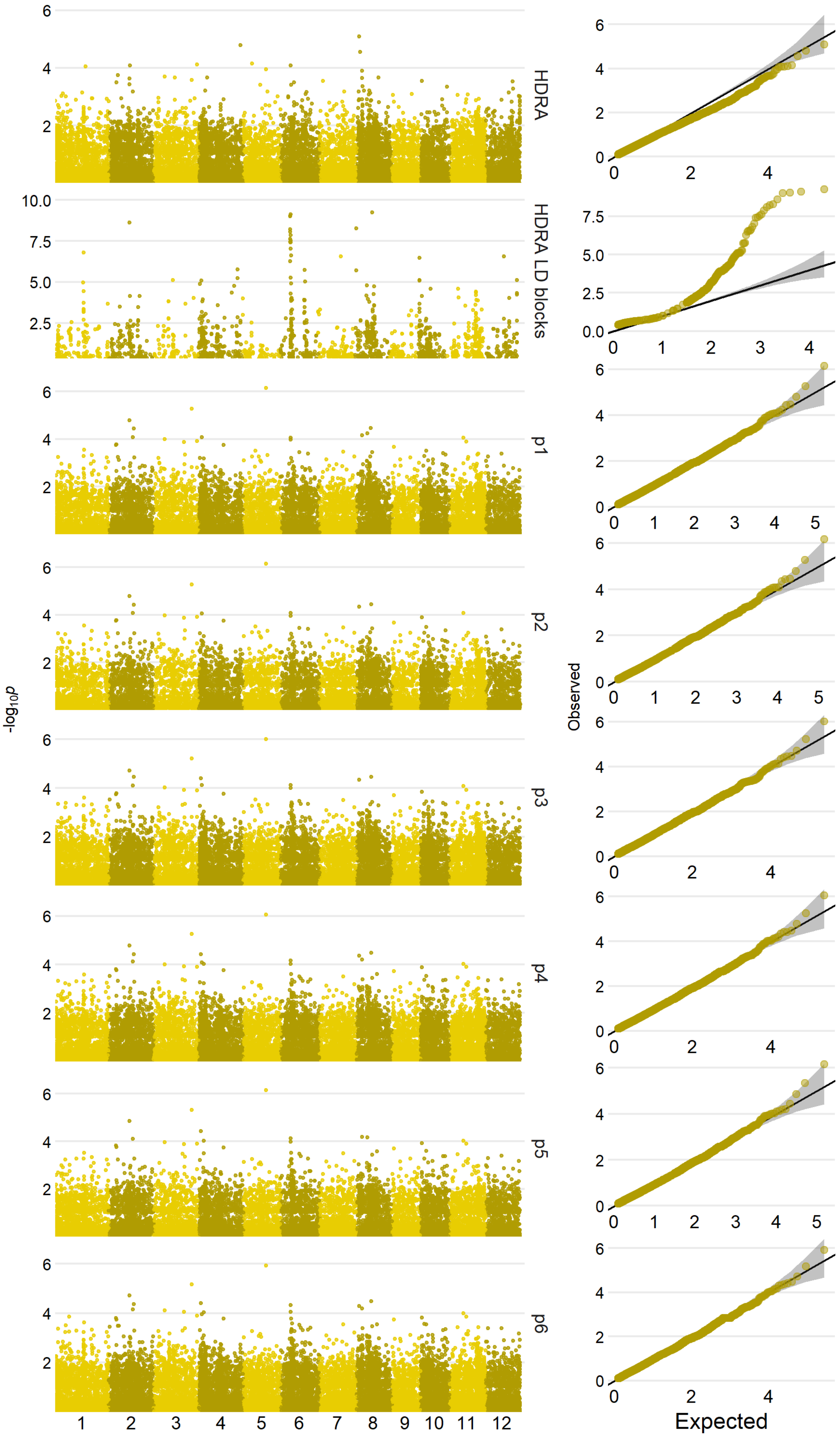

Supplemental Figure 19. Genome-wide association analysis using the HDRA SNPs, HDRA LD blocks, or pruned sets of the RICE-RP SNPs (p1-p6) for *tej* RHL. Manhattan plots are in the left column and their corresponding QQ plots are in the right column.

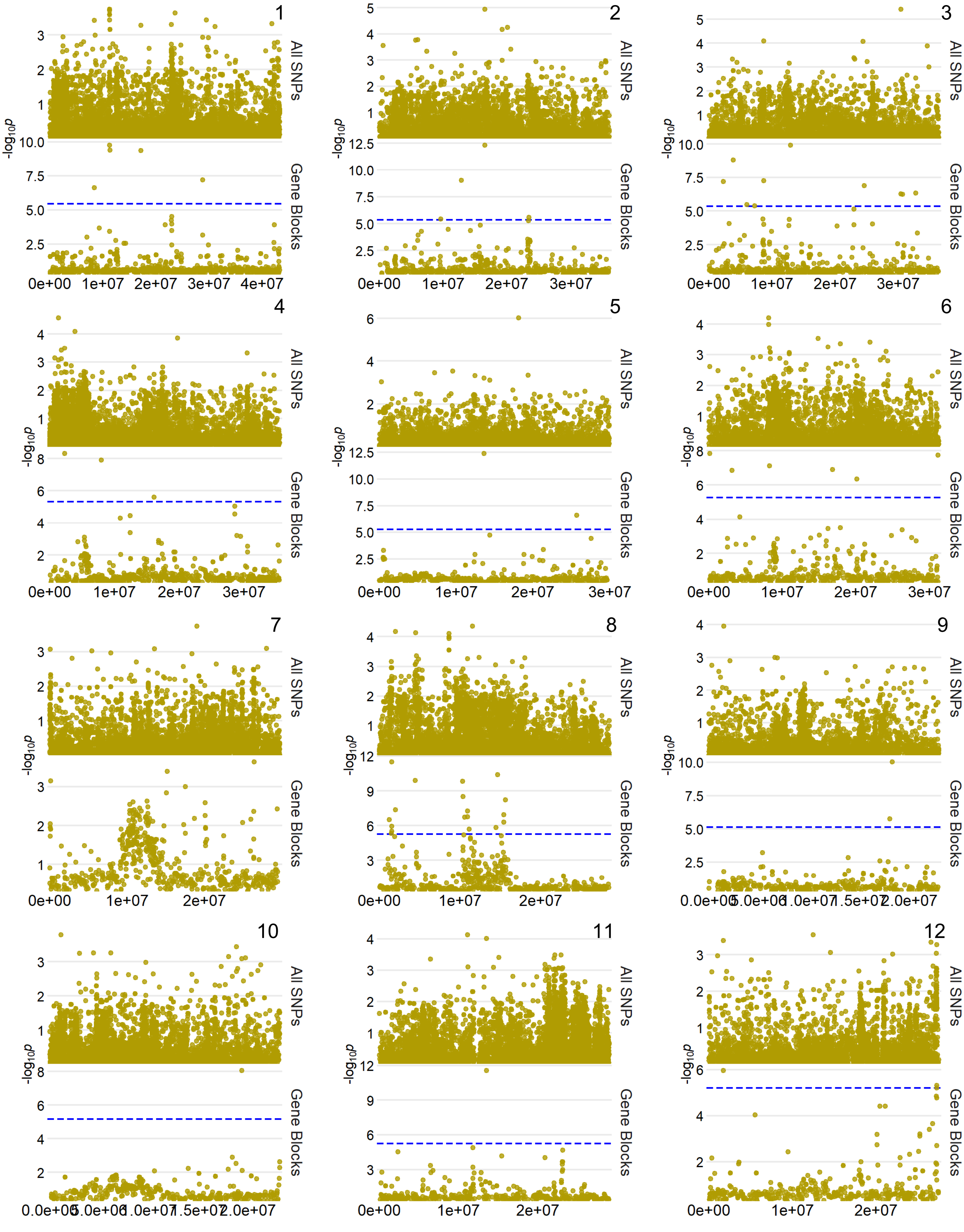

Supplemental Figure 20 *tej* RHL Chromosome-wide association analysis using the RICE-RP SNPs (upper) or the SNPs collapsed into gene blocks (lower) for chromosomes 1-12, indicated in the upper right. Blue lines indicate significance at the Bonferroni adjusted p-value < 0.05 for individual SNPs and 0.01 for gene blocks.

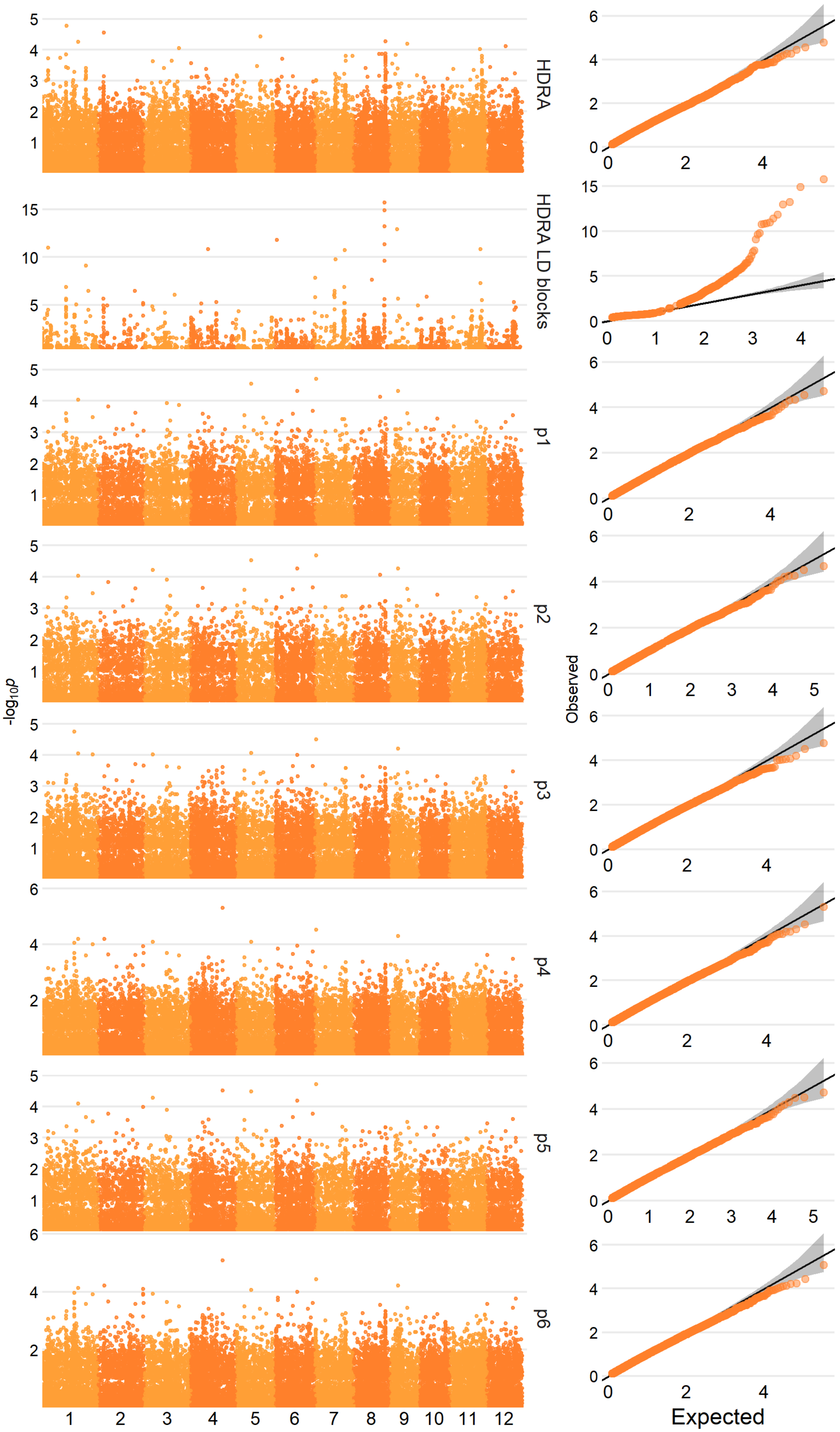

Supplemental Figure 21. Genome-wide association analysis using the HDRA SNPs, HDRA LD blocks, or pruned sets of the RICE-RP SNPs (p1-p6) for *trj* RHD. Manhattan plots are in the left column and their corresponding QQ plots are in the right column.

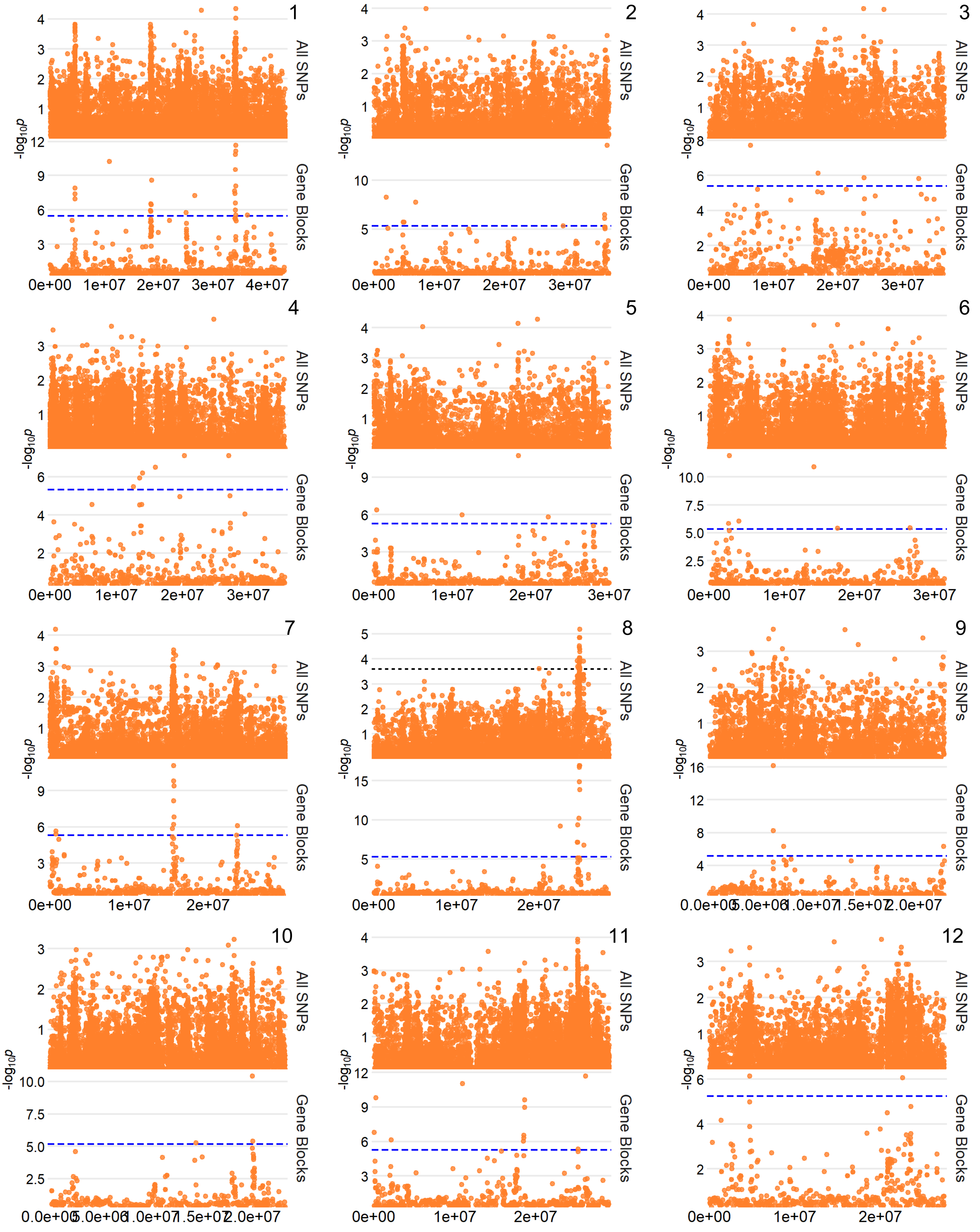

Supplemental Figure 22 *trj* RHD Chromosome-wide association analysis using the RICE-RP SNPs (upper) or the SNPs collapsed into gene blocks (lower) for chromosomes 1-12, indicated in the upper right. Blue lines indicate significance at the Bonferroni adjusted p-value < 0.05 for individual SNPs and 0.01 for gene blocks.

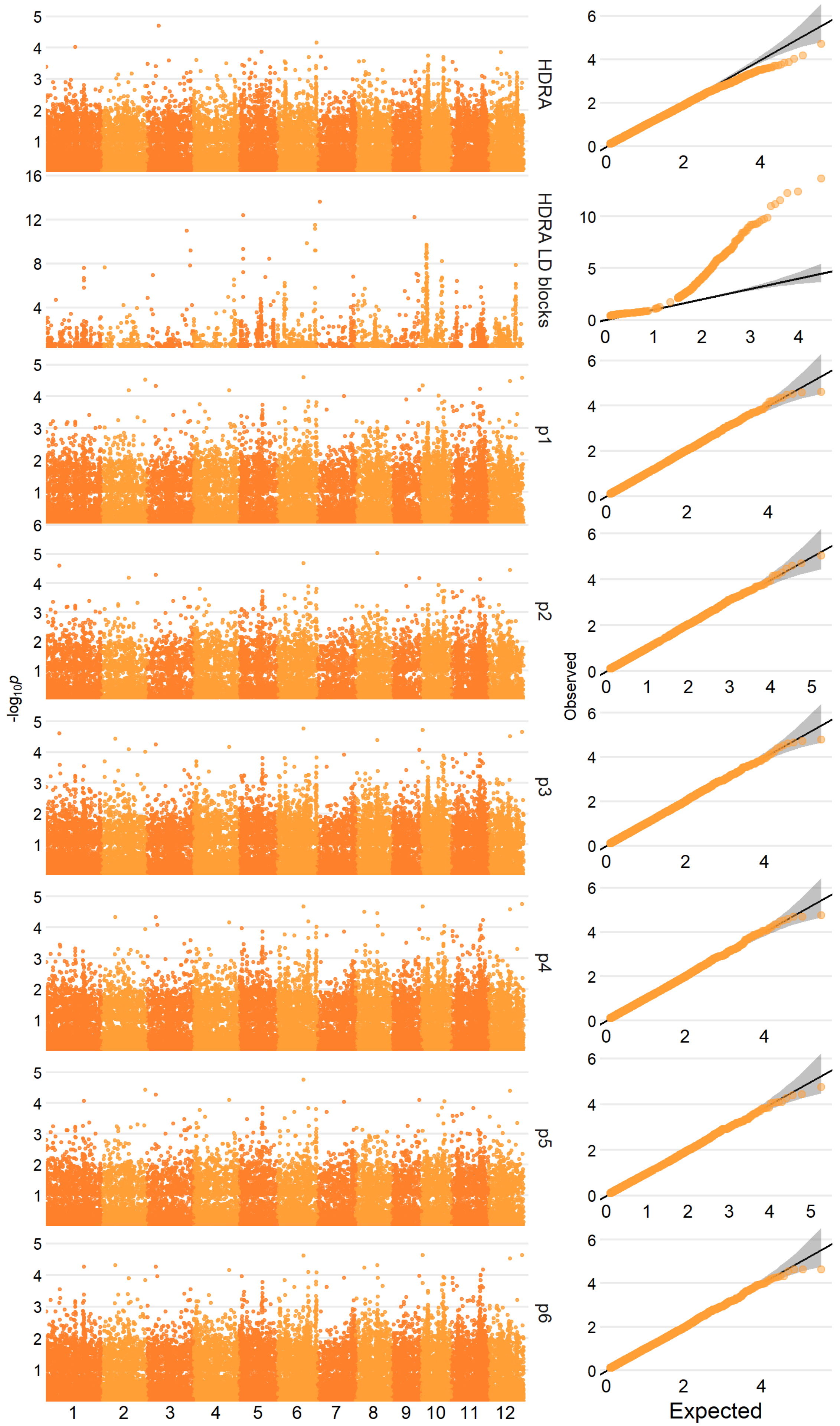

Supplemental Figure 23. Genome-wide association analysis using the HDRA SNPs, HDRA LD blocks, or pruned sets of the RICE-RP SNPs (p1-p6) for *trj* RHD. Manhattan plots are in the left column and their corresponding QQ plots are in the right column.

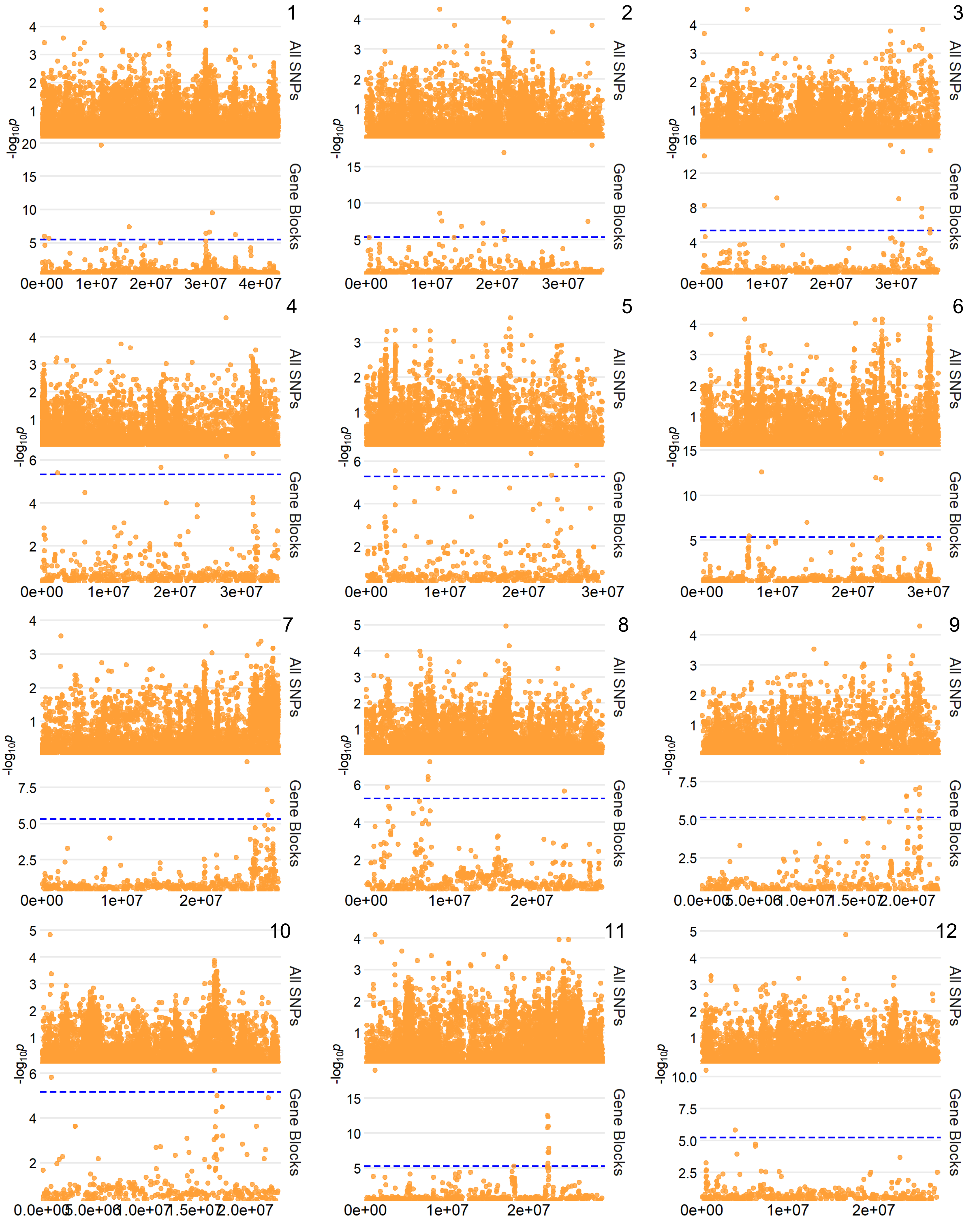

Supplemental Figure 24 *trj* RHL Chromosome-wide association analysis using the RICE-RP SNPs (upper) or the SNPs collapsed into gene blocks (lower) for chromosomes 1-12, indicated in the upper right. Blue lines indicate significance at the Bonferroni adjusted p-value < 0.05 for individual SNPs and 0.01 for gene blocks.

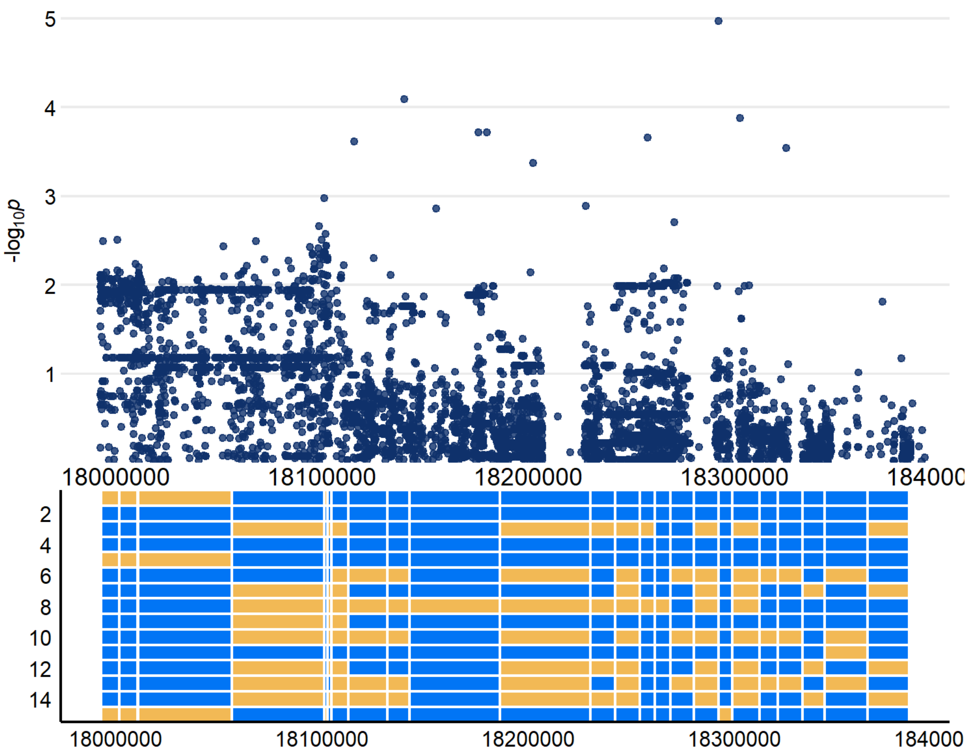

Supplemental Figure 25 Haplotype exploration of the region on chromosome 5 associated with RHL in *ALL.* Due to the large number of haplotypes across this region, we did not estimate trait values in relation to the haplotypes.

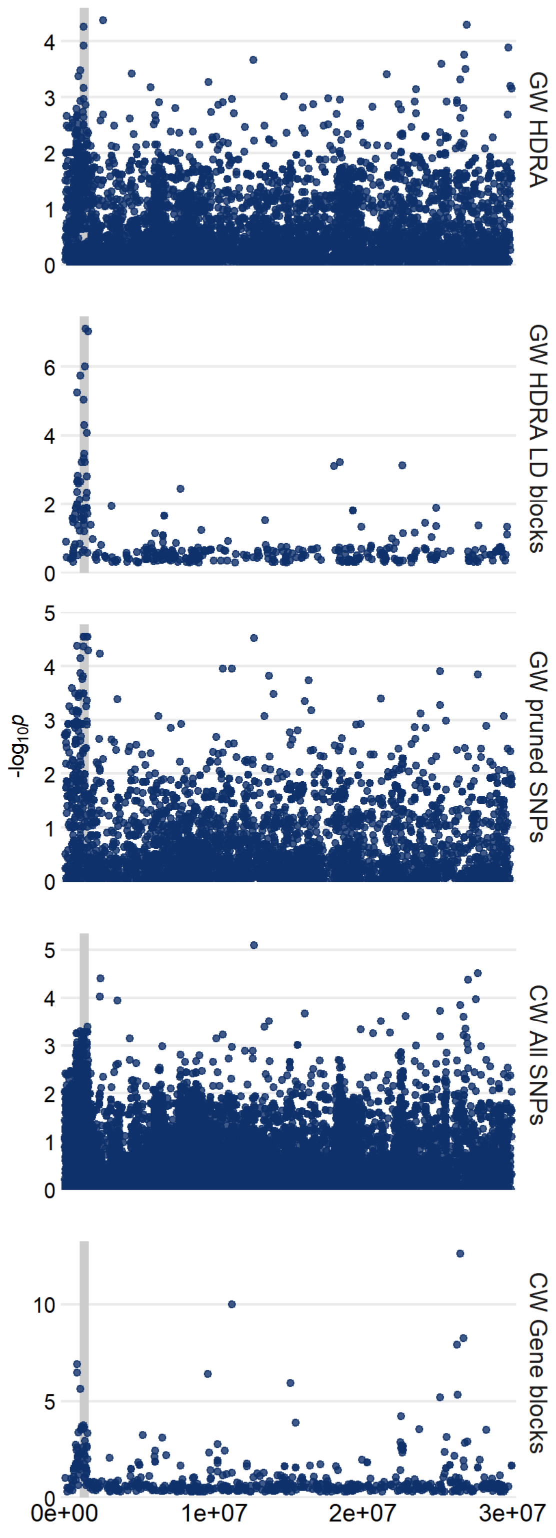

Supplemental Figure 26 Region D4 from *ind* for RHD on chromosome 5 lacks support across multiple association runs.

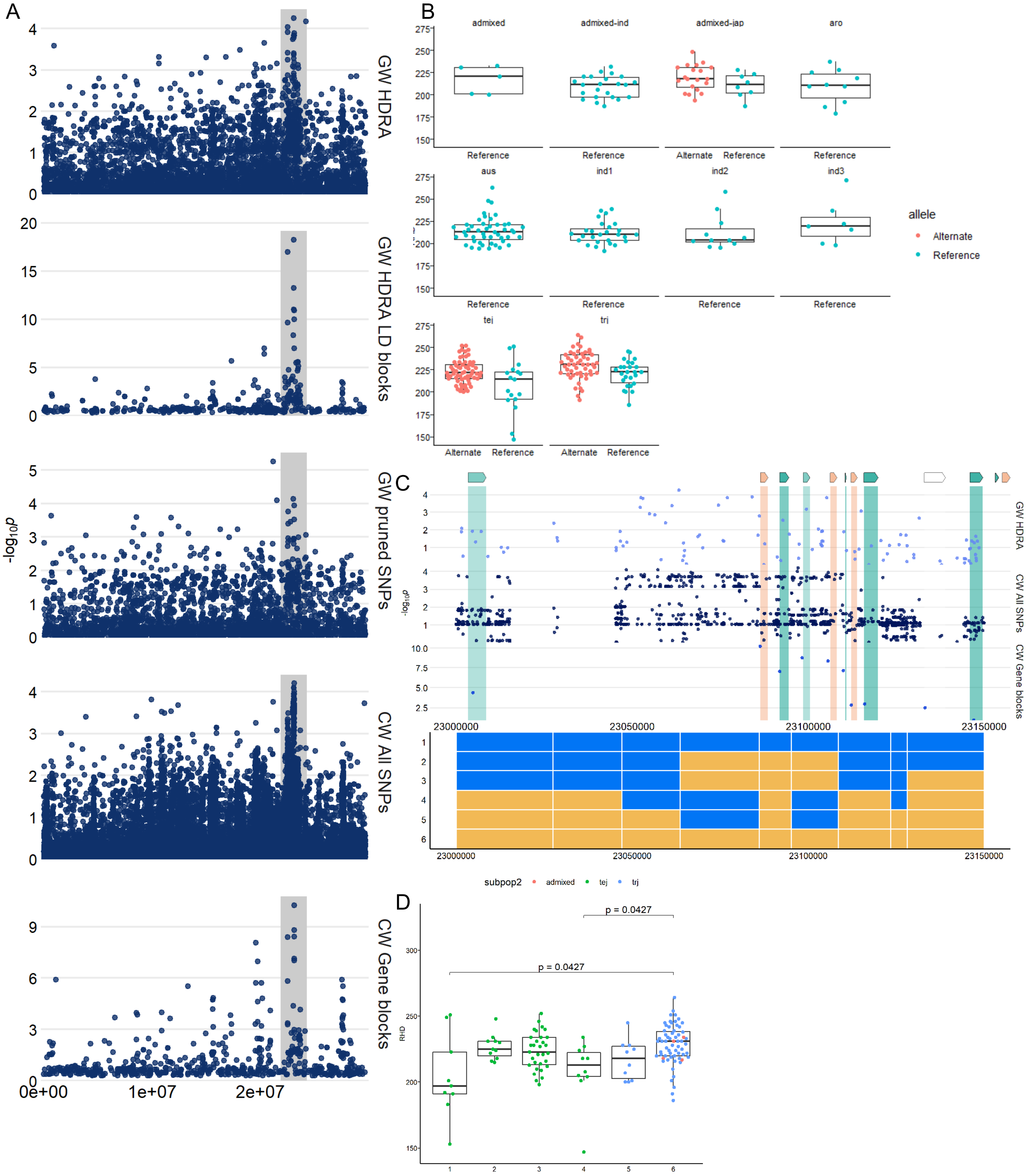

Supplemental Figure 27 Region D7 associated with RHD from *ALL* and *JAPONICA* lines. The association is largely driven by differences in phenotype at the clade-level. A) From top-to-bottom, genome-wide (GW) HDRA, Genome-wide HDRA LD blocks, GW pruned SNPs from the RICE-RP (single representative), chromosome-wide all RICE-RP SNPs, and chromosome-wide gene blocks from the RICE-RP SNPs with the associated genomic region in gray. B) RHD phenotypes of individuals carrying the reference or alternate allele at the MS-SNP split by subpopulation. CMagnification of the GW-HDRA, RICE-RP chromosome-wide, and CW-gene block Manhattan plots of the genomic region. Genes are overlaid on this plot and colored as follows. Orange genes are not expressed in root hairs or root-hair forming cells according to Huang et al. (2017). Below, haplotypes 1-6 are displayed with blue representing the reference identity and yellow, alternate, across this region. D) Phenotypes of the haplotypes with significance indicated as a BH-adjusted, Kruskal-Wallace test with a Dunn posthoc test

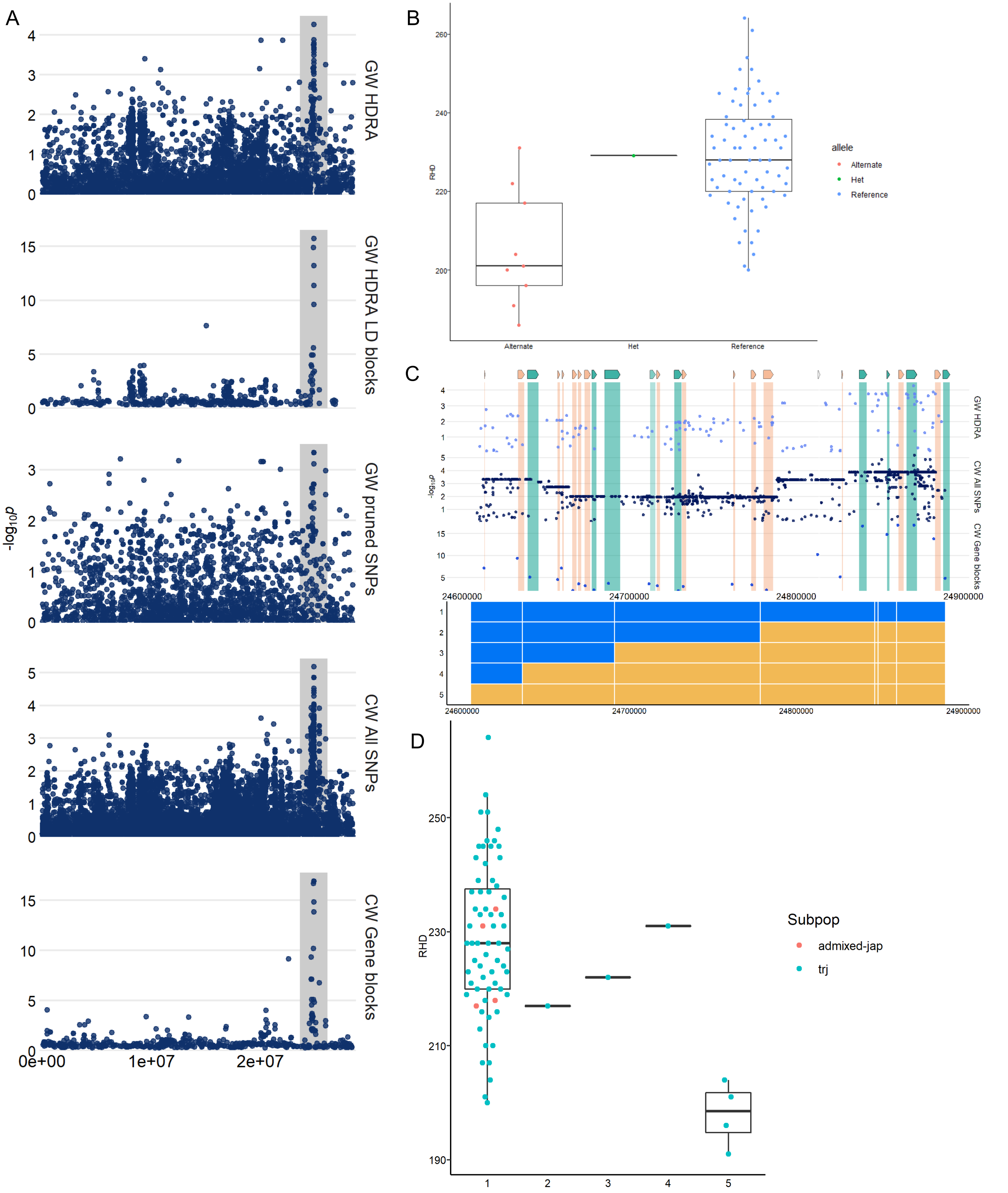

Supplemental Figure 28 Region D9 associated with *RHD* in *trj* on chromosome 8. The association is largely driven by differences in phenotype at the clade-level. A) From top-to-bottom, genome-wide (GW) HDRA, Genome-wide HDRA LD blocks, GW pruned SNPs from the RICE-RP (single representative), chromosome-wide all RICE-RP SNPs, and chromosome-wide gene blocks from the RICE-RP SNPs with the associated genomic region in gray. B) RHD phenotypes of individuals carrying the reference or alternate allele at the MS-SNP. C) Magnification of the GW-HDRA, RICE-RP chromosome-wide, and CW-gene block Manhattan plots of the genomic region. Genes are overlaid on this plot and colored as follows. Orange genes are not expressed in root hairs or root-hair forming cells according to Huang et al. (2017). Below, haplotypes 1-6 are displayed with blue representing the reference identity and yellow, alternate, across this region. D) Phenotypes of the haplotypes.

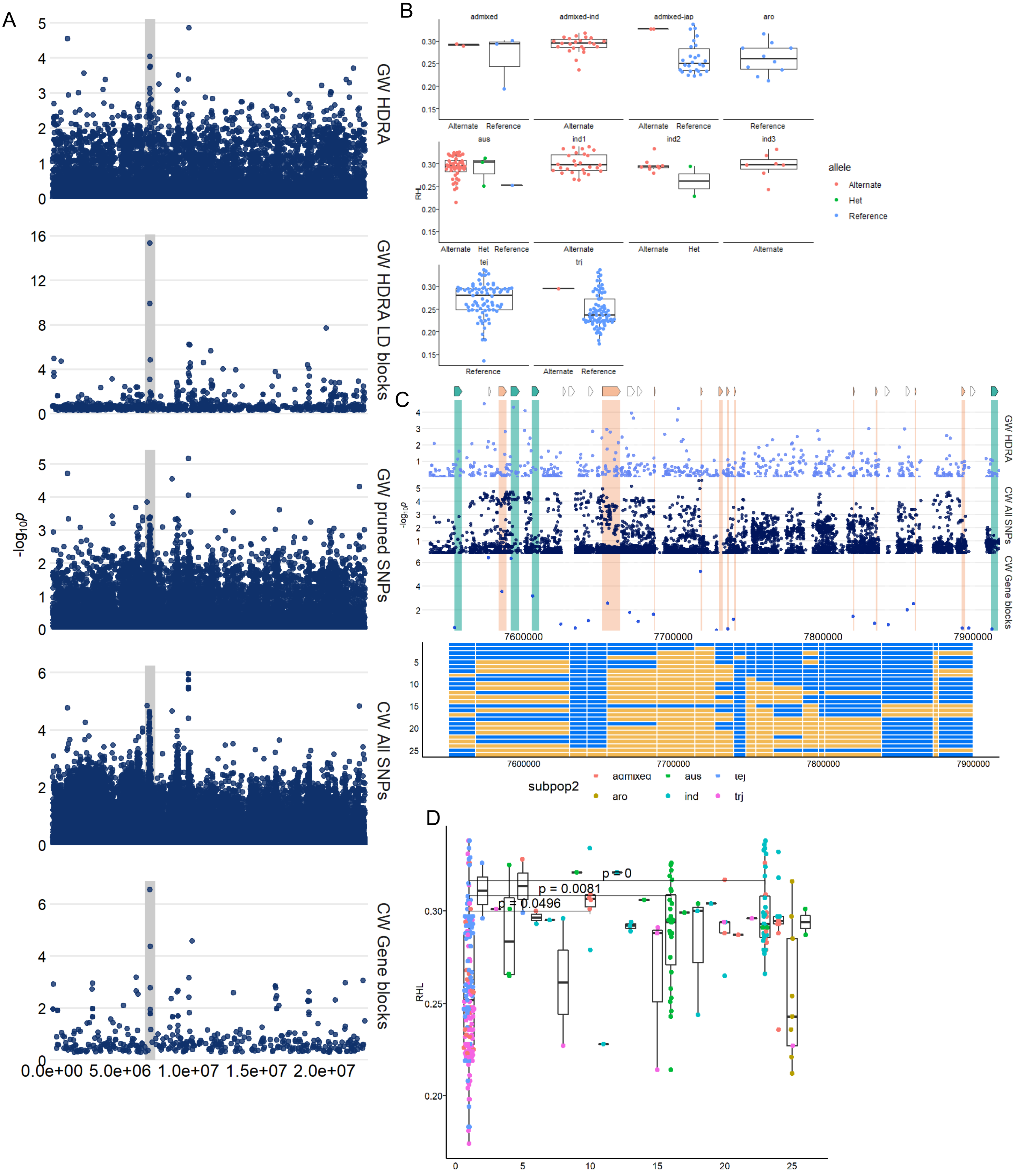

Supplemental Figure 29 Region D10 associated with *RHD* in *ALL* on chromosome 9 A) From top-to-bottom, genome-wide (GW) HDRA, Genome-wide HDRA LD blocks, GW pruned SNPs from the RICE-RP (single representative), chromosome-wide all RICE-RP SNPs, and chromosome-wide gene blocks from the RICE-RP SNPs with the associated genomic region in gray. B) RHD phenotypes of individuals carrying the reference or alternate allele at the MS-SNP split by subpopulation. C) Magnification of the GW-HDRA, RICE-RP chromosome-wide, and CW-gene block Manhattan plots of the genomic region. Genes are overlaid on this plot and colored as follows. Orange genes are not expressed in root hairs or root-hair forming cells according to Huang et al. (2017). Below, haplotypes are displayed with blue representing the reference identity and yellow, alternate, across this region. D) Phenotypes of the haplotypes with significance indicated as a BH-adjusted, Kruskal-Wallace test with a Dunn posthoc test

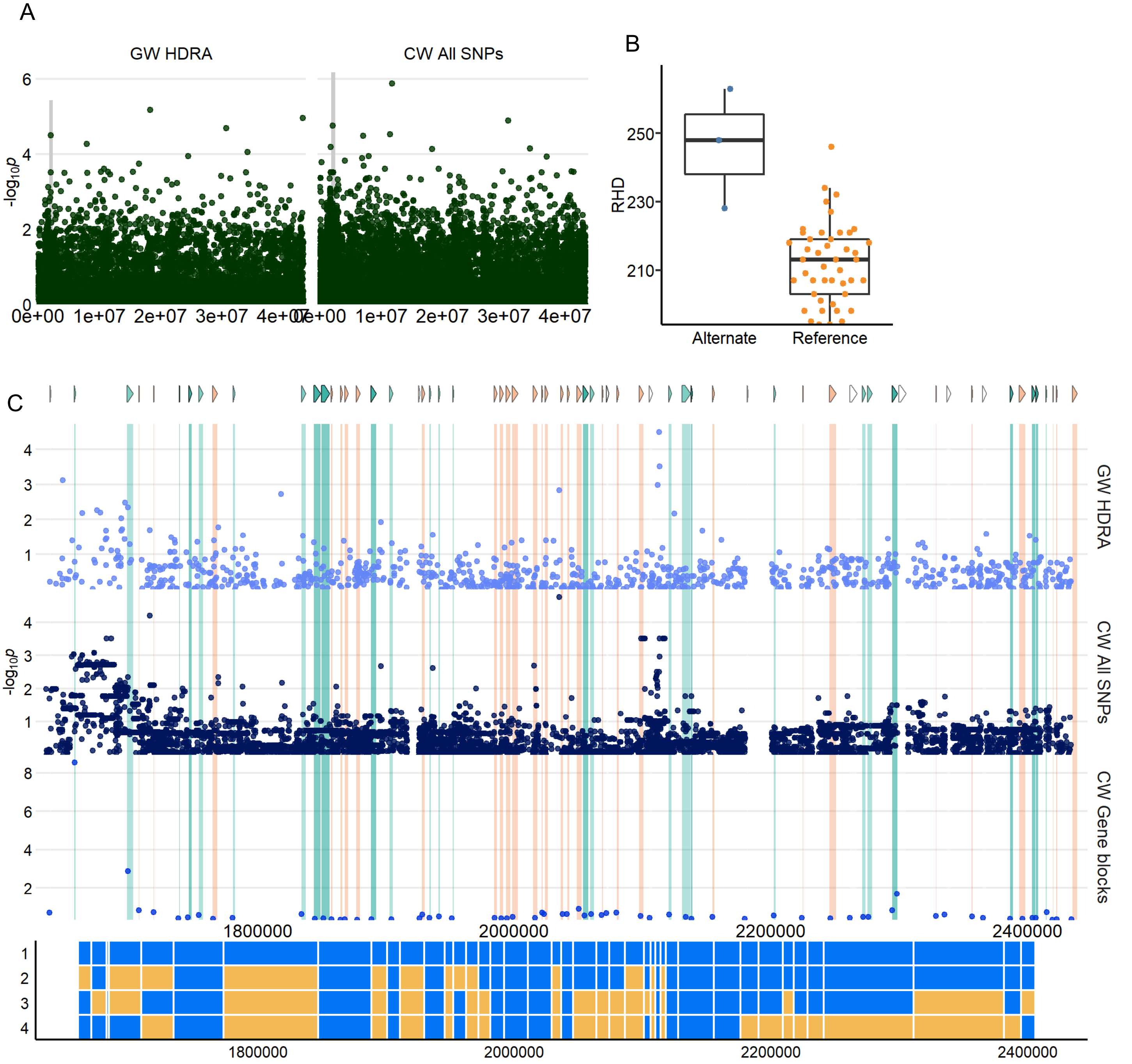

Supplemental Figure 30 Haplotype exploration of the region on chromosome 1 associated with RHD in *aus.* A Genome-wide (GW) HDRA (left) and chromosome-wide all RICE-RP SNPs (right) with the associated genomic region in gray. B) RHD phenotypes of individuals carrying the reference or alternate allele at the MS-SNP. C) Magnification of the GW-HDRA, RICE-RP chromosome-wide, and CW-gene block Manhattan plots o the genomic region. Genes are overlaid on this plot and colored as follows. Orange genes are not expressed in root hairs or root-hair forming cells according to Huang et al. (2017). Below, haplotypes 1-4

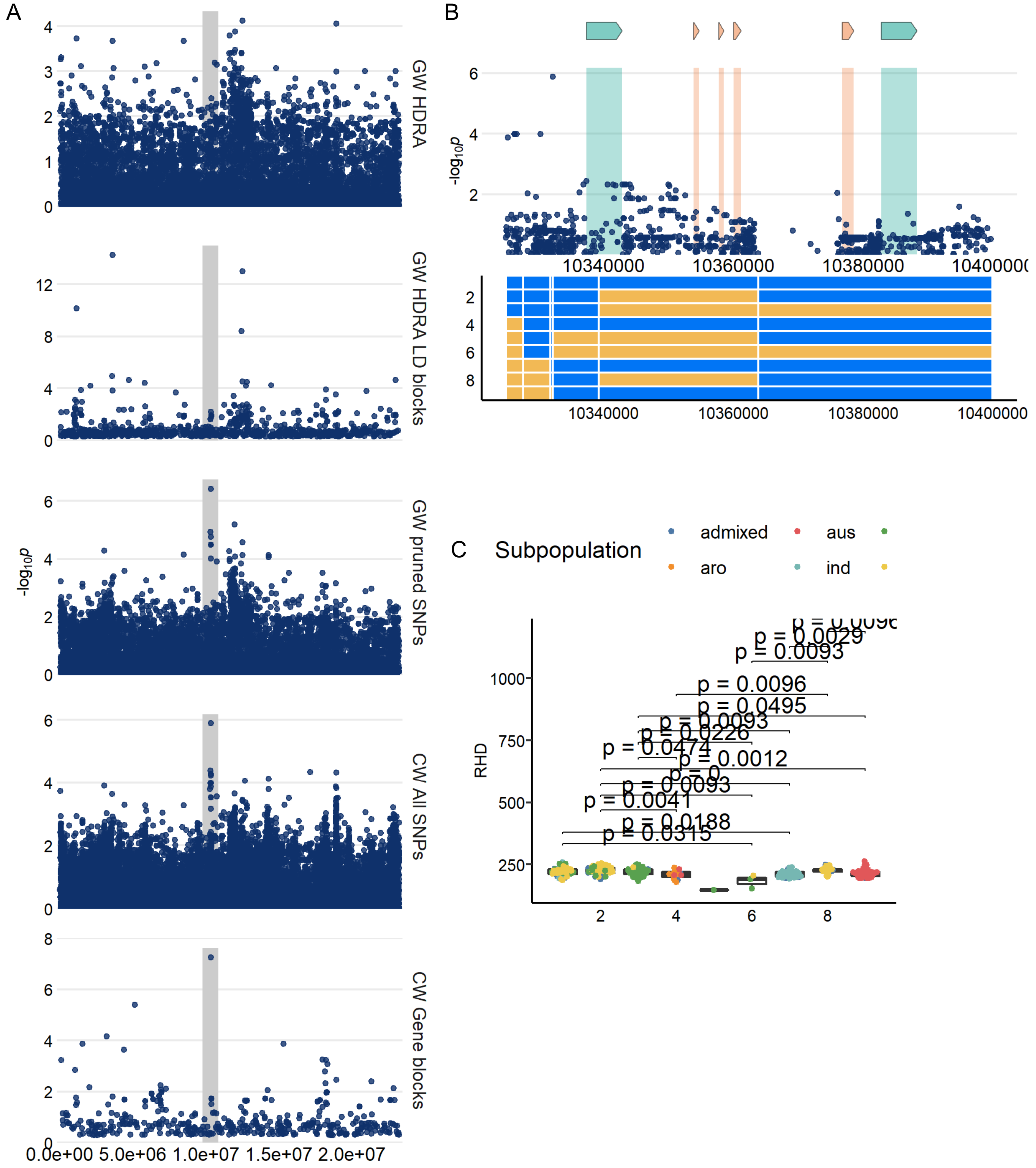

Supplemental Figure 31 Haplotype exploration of the region on chromosome 10 associated with RHD in *ALL* and *JAPONICA.* A) From top-to-bottom, genome-wide (GW) HDRA, Genome-wide HDRA LD blocks, GW pruned SNPs from the RICE-RP (single representative), chromosome-wide all RICE-RP SNPs, and chromosome-wide gene blocks from the RICE-RP SNPs with the associated genomic region in gray. B) RHD phenotypes of individuals carrying the reference or alternate allele at the MS-SNP split by subpopulation. C) Magnification of the RICE-RP chromosome-wide Manhattan plot of the genomic region. Genes are overlaid on this plot and colored as follows. Orange genes are not expressed in root hairs or root-hair forming cells according to Huang et al. (2017). Below, haplotypes are displayed with blue representing the reference identity and yellow, alternate, across this region. D) Phenotypes of the haplotypes with significance indicated as a BH-adjusted, Kruskal-Wallace test with a Dunn posthoc test

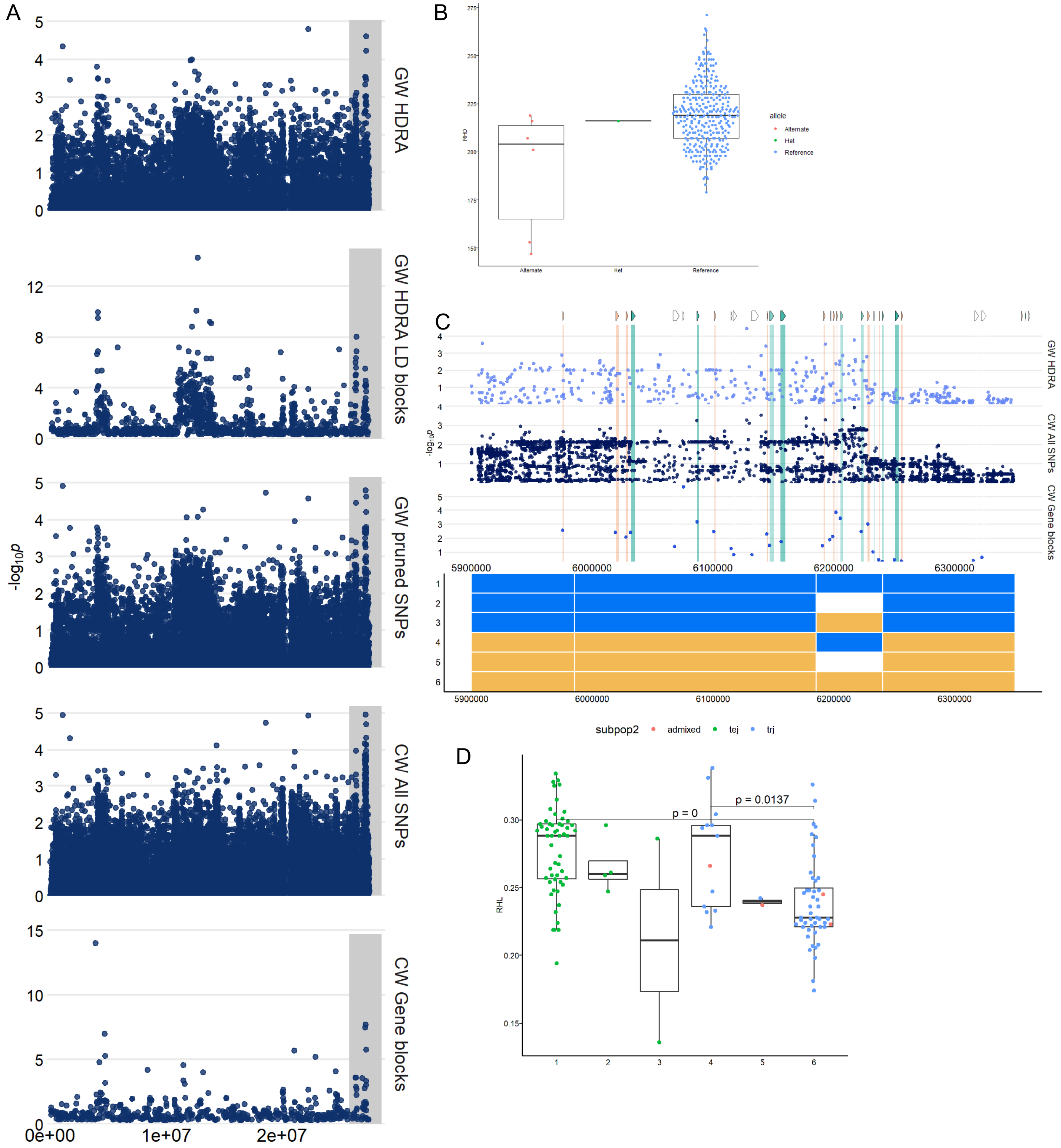

Supplemental Figure 32 Haplotype exploration of region D14 on chromosome 12 associated with RHD in *ALL* and *JAPONICA*. A) From top-to-bottom, genome-wide (GW) HDRA, Genome-wide HDRA LD blocks, GW pruned SNPs from the RICE-RP (single representative), chromosome-wide all RICE-RP SNPs, and chromosome-wide gene blocks from the RICE-RP SNPs with the associated genomic region in gray. B) RHD phenotypes of individuals carrying the reference or alternate allele at the MS-SNP split by subpopulation. C) Magnification of the GW-HDRA, RICE-RP chromosome-wide, and CW-gene block Manhattan plots of the genomic region. Genes are overlaid on this plot and colored as follows. Orange genes are not expressed in root hairs or root-hair forming cells according to Huang et al. (2017). Below, haplotypes are displayed with blue representing the reference identity and yellow, alternate, across this region. D) Phenotypes of the haplotypes with significance indicated as a BH-adjusted, Kruskal-Wallace test with a Dunn posthoc test
